# RBM12 Maintains Glioma Stem Cells by Activating Amino Acid‐Dependent mTORC1 Signaling via *SLC7A5* mRNA Stabilization

**DOI:** 10.1002/advs.76239

**Published:** 2026-06-22

**Authors:** Hong Lei, Wenlong Luo, Shu Zhou, Lihao Wan, Peng Ling, Zhihua Huang, Zhihua Qian, Chenfei Lu, Mengyue Guo, Zhen Xue, Jun Qin, Ningwei Zhao, Jianghong Man, Wenchao Zhou, Zhiqiang Dong, Shutong Xu, Zhipeng Zhou, Xiuxing Wang, Weiwei Tao

**Affiliations:** ^1^ College of Biomedicine and Health College of Life Science and Technology Huazhong Agricultural University Wuhan Hubei China; ^2^ National Health Commission Key Laboratory of Antibody Techniques Department of Cell Biology School of Basic Medical Sciences Nanjing Medical University Nanjing Jiangsu China; ^3^ Department of Neurosurgery The First Affiliated Hospital of Nanjing Medical University Nanjing Medical University Nanjing Jiangsu China; ^4^ Hubei Provincial Clinical Research Center of Central Nervous System Repair and Functional Reconstruction Taihe Hospital Hubei University of Medicine Shiyan Hubei China; ^5^ Affiliated Hospital of Nanjing University of Chinese Medicine Nanjing University of Chinese Medicine Nanjing Jiangsu China; ^6^ China Exposomics Institute Shanghai China; ^7^ National Center of Biomedical Analysis Beijing China; ^8^ Department of Pathology The First Affiliated Hospital of USTC Division of Life Sciences and Medicine University of Science and Technology of China Hefei Anhui China

**Keywords:** amino acid metabolism, glioblastoma, glioma stem cells, mTORC1 pathway, RBM12, SLC7A5

## Abstract

Reprogramming of amino acid metabolism is crucial for the rapid proliferation of cancer cells, including cancer stem cells. However, the molecular mechanisms underlying this reprogramming in glioma stem cells (GSCs) remain poorly understood. Here, we report that the RNA‐binding protein RBM12 increases the intracellular levels of large neutral amino acids, thereby activating the mTORC1 pathway and promoting GSC proliferation, self‐renewal, and glioblastoma (GBM) growth. Mechanistically, RBM12 stabilizes the mRNA of the amino acid transporter *SLC7A5*, thereby increasing intracellular levels of large neutral amino acids, which subsequently activates the mTORC1 pathway. Further studies reveal that RBM12 enhances *SLC7A5* mRNA stability by recruiting ALKBH5 to remove m^6^A modifications on *SLC7A5* mRNA. Importantly, pharmacological inhibition of the RBM12‐SLC7A5 axis using the SLC7A5 inhibitor JPH203 effectively suppresses GBM growth. These findings elucidate a novel role for RBM12‐SLC7A5 signaling in the malignant growth of GBM and highlight the therapeutic potential of targeting this axis for GBM treatment.

## Graphical abstract

This study shows that in glioma stem cells (GSCs), RBM12 recruits ALKBH5 to remove m^6^A from *SLC7A5* transcripts, thereby enhancing mRNA stability, which elevates large neutral amino acid (LNAA) levels and activates mTORC1, promoting GSC proliferation, self‐renewal, and tumor growth. Pharmacological inhibition of this axis with JPH203 suppresses GSC maintenance and tumor progression. Created in BioRender. Lei, H. (2026) https://BioRender.com/geapgqj.

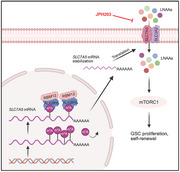

## 1 Introduction

Glioblastoma (GBM) is the most common and malignant type of primary brain tumor in adults [1, 2, 3]. Patients diagnosed with GBM typically have a median survival of 14 to 24 months and a less than 5% chance of surviving beyond 5 years [2, 4]. The poor prognosis of GBM patients is largely attributed to limited therapeutic options, poor treatment response, and a high rate of disease recurrence [5]. GBM is characterized by its cellular heterogeneity and hierarchical structure, which includes a subpopulation of stem cell‐like cancer cells known as glioma stem cells (GSCs) at the apex of the differentiation hierarchy [5, 6]. These GSCs exhibit an exceptional capacity for rapid proliferation and self‐renewal, which are intricately linked to tumor initiation, sustained growth, and recurrence [7, 8]. Therefore, a better understanding of the molecular mechanisms driving the proliferation and self‐renewal of GSCs is crucial for developing targeted therapeutic strategies against these cells, which may significantly improve the efficacy of GBM treatment.

Unlike normal cells, cancer cells undergo metabolic reprogramming, a process in which they alter their metabolic pathways to support their rapid proliferation [9, 10]. Among the diverse metabolic alterations, the dysregulation of amino acid metabolism has garnered increasing attention due to its profound impact on tumor progression. This dysregulation is characterized by abnormal changes in transport, synthesis, and catabolism of amino acids [11]. For instance, liver cancer cells upregulate the expression of the SLC7A family of arginine transporters to enhance arginine uptake, thereby promoting tumor growth [12]. Another study found that these cells enhance the activity of PHGDH, a key enzyme in serine synthesis, which also contributes to liver tumor growth [13]. Additionally, ovarian cancer cells with low FAH expression, an enzyme that catalyzes the final step of tyrosine catabolism, exhibit decreased chemosensitivity and enhanced survival [14]. Therefore, reprogramming of amino acid metabolism is essential for maintaining cancer cell function and represents a potential therapeutic target. However, the molecular mechanisms underlying amino acid metabolic reprogramming in GSCs remain poorly understood.

RNA‐binding proteins (RBPs) are defined by their RNA‐binding domains, which enable them to interact with RNA [15, 16, 17]. RBPs play crucial roles in regulating various aspects of RNA metabolism, including RNA processing, transport, translation, and stability [18]. Given their pivotal role in the RNA lifecycle, it is not surprising that dysregulation of RBPs can lead to various diseases, including cancer [19, 20]. RNA‐binding motif proteins (RBMs) are an important family of RBPs that contain at least one RNA recognition motif (RRM) domain. Accumulating studies have demonstrated that RBMs are abnormally expressed in various types of cancer and contribute to tumorigenesis and progression [21]. For instance, RBMS1 is upregulated in lung cancer and inhibits ferroptosis by facilitating the translation of *SLC7A11*, thereby promoting tumor progression [22]. In laryngeal cancer, RBM15 is significantly increased and promotes tumor progression through IGF2BP3‐mediated stabilization of *TMBIM6* mRNA [23]. In head‐neck squamous cell carcinoma, RBM33 is highly expressed and promotes tumorigenesis by stabilizing *DDIT4* mRNA [24]. Despite the growing evidence of RBMs' involvement in various cancers, their expression and roles in GBM, especially in GSCs, remain largely unexplored. Moreover, it is also unclear whether RBMs can regulate GSC metabolism to sustain their function.

To interrogate the potential relationship between RBM expression and GSC maintenance, we performed an integrative analysis of two microarray datasets from GEO and a clinical database, identifying *RBM12* as the sole *RBM* gene enriched in GBM and GSCs. RBM12 is a member of the RBM family and is composed of five RRMs, two proline‐rich regions, and several putative transmembrane domains [25]. Recent studies have demonstrated that RBM12 is significantly upregulated in hepatocellular carcinoma and contributes to tumor progression [26, 27]. However, it remains unclear whether RBM12 is associated with the occurrence and development of other tumors. Here, we analyzed the expression of RBM12 in GBM and found that RBM12 is preferentially expressed in GSCs and is induced by the transcription factor OTX1. We demonstrated that RBM12 stabilizes the mRNA of the amino acid transporter *SLC7A5*, thereby increasing intracellular levels of large neutral amino acids and downstream mTORC1 signaling, which in turn promotes GSC proliferation, self‐renewal, and tumor growth. We further demonstrated that RBM12 enhances *SLC7A5* mRNA stability by recruiting ALKBH5 to remove m^6^A modifications on *SLC7A5* mRNA. Importantly, pharmacological inhibition of the RBM12‐SLC7A5 axis with the SLC7A5 inhibitor JPH203 suppressed GSC proliferation, self‐renewal, and tumor growth, suggesting that targeting this signaling axis may be an effective therapeutic strategy against GBM.

## 2 Results

### 2.1 RBM12 is Preferentially Expressed in GSCs

**FIGURE 1:**
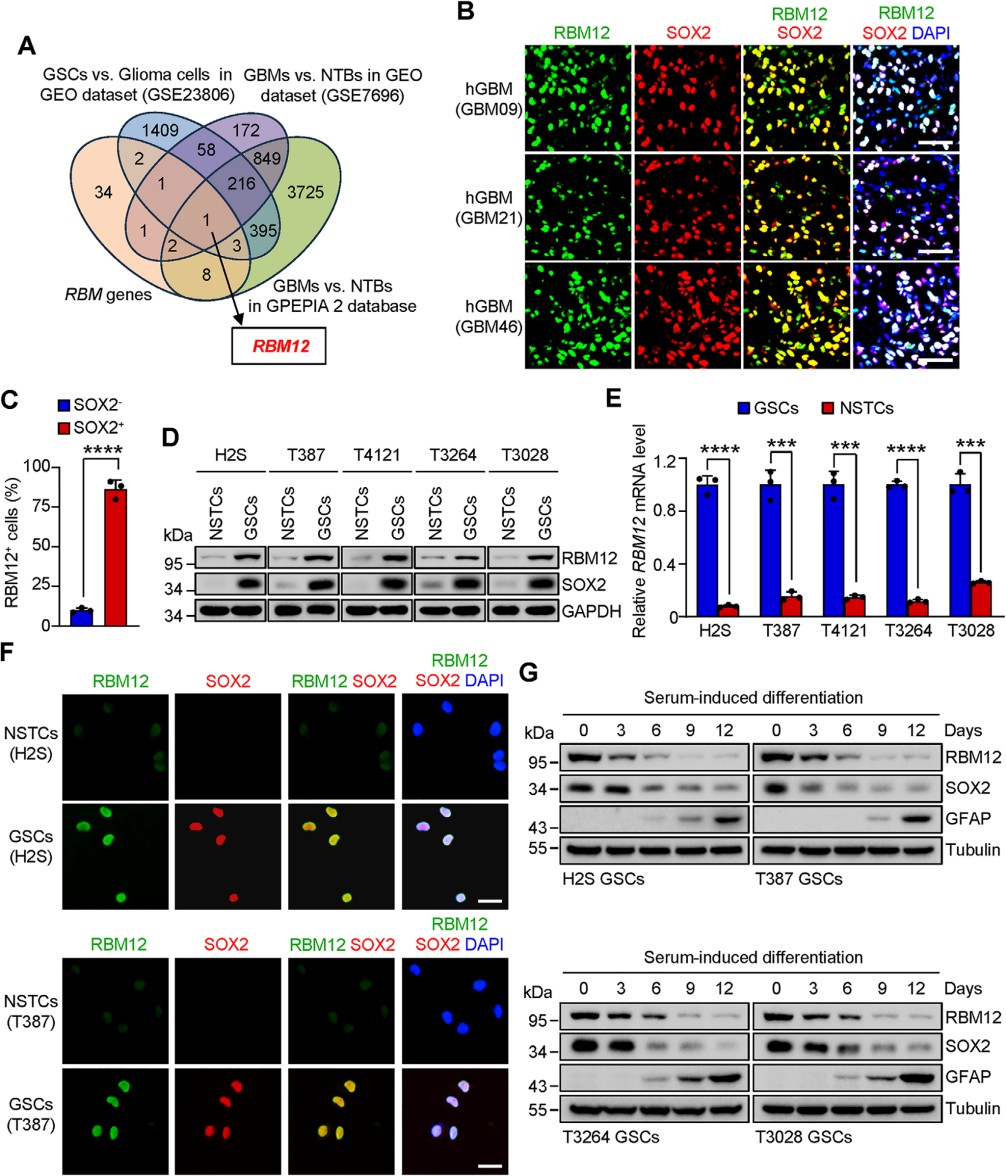
RBM12 is preferentially expressed by GSCs. (A) Venn diagram showing the overlap among the following four datasets: genes significantly upregulated in GSCs compared to glioma cells in the GEO dataset GSE23806 (fold change > 2, *p* < 0.05), genes significantly upregulated in GBM compared to NTBs in the GEO dataset GSE7696 (fold change > 2, *p* < 0.05), genes significantly upregulated in GBM compared to NTBs in the GEPIA 2 database (fold change > 2, *p* < 0.05), and *RBM* genes. (B) Immunofluorescent staining of RBM12 (green) and SOX2 (red) in human GBM tissues. Scale Bar, 50 µm. (C) Quantification of the fraction of RBM12^+^ cells in SOX2^−^ and SOX2^+^ populations in three independent human GBM tissues. *n* = 3. (D) Immunoblot analysis of RBM12 and SOX2 expression in five pairs of matched GSCs and NSTCs. (E) qPCR analysis of *RBM12* mRNA expression in five pairs of matched GSCs and NSTCs. *n* = 3. (F) Immunofluorescent staining of RBM12 (green) and SOX2 (red) in GSCs and matched NSTCs. Scale Bar, 30 µm. (G) Immunoblot analysis of RBM12, SOX2, and GFAP expression during serum‐induced GSC differentiation. Data information: Data are presented as mean ± SD. ^***^ *P* < 0.001, ^****^ *P* < 0.0001, two‐tailed unpaired *t*‐test (C and E).

To identify the potential *RNA‐binding motif* (*RBM*) genes that are preferentially expressed in GSCs, we performed an integrative analysis leveraging multiple datasets. These datasets included the GEPIA 2 dataset, which encompasses gene profiles of 163 GBMs and 207 non‐tumor brains (NTBs); microarray data from GSE7696, comprising 80 GBMs and 4 NTBs; and microarray data from GSE23806, featuring 27 GSCs and 32 glioma cells. Our meticulous analysis identified *RBM12* as the sole *RBM* gene enriched in GBM and GSCs (Figure 1A). Subsequently, we analyzed the expression of *RBM12* in GBMs and NTBs by using the TCGA HG‐U133A, TCGA RNA‐seq, Rembrandt, Murat, and Kamoun datasets. These analyses confirmed that the mRNA expression of *RBM12* was significantly elevated in GBM tissues compared to NTBs (Figure S1A–E). In addition, we found that the protein levels of RBM12 were much higher in GBMs compared to NTBs, as determined using the CPTAC database (Figure S1F). To confirm the enrichment of RBM12 in GSCs, we analyzed the expression patterns of RBM12 and the GSC marker SOX2 within human primary GBM specimens. Notably, immunofluorescent staining revealed a pronounced co‐expression of RBM12 with SOX2 in these samples (Figure 1B,C). To further substantiate RBM12's preferential expression in GSCs, we examined its expression levels in isolated GSCs and paired non‐stem tumor cells (NSTCs) that were functionally authenticated, as described in the Methods section. Immunoblot and qPCR analyses detected much higher protein and mRNA levels of *RBM12* in GSCs relative to NSTCs (Figure 1D,E). Furthermore, immunofluorescent staining of RBM12 and the GSC marker SOX2 in matched GSCs and NSTCs corroborated RBM12's preferential expression in GSCs (Figure 1F). As the GSC population decreases after differentiation, we examined the expression of RBM12 throughout the process of serum‐induced differentiation in GSCs. Immunoblot analysis indicated a progressive decline in both RBM12 and SOX2 levels during this process, concomitant with an upregulation of the differentiation marker GFAP (Figure 1G). We further compared RBM12 expression between GSCs and neural progenitor cells (NPCs), and the results showed that RBM12 was expressed at substantially higher levels in GSCs than in NPCs (Figure S1G). Collectively, these data demonstrate that RBM12 is preferentially expressed in GSCs, suggesting a potential role of RBM12 in GSC maintenance.

### 2.2 RBM12 Promotes GSC Proliferation, Self‐Renewal, and GSC‐Driven Tumor Growth

**FIGURE 2:**
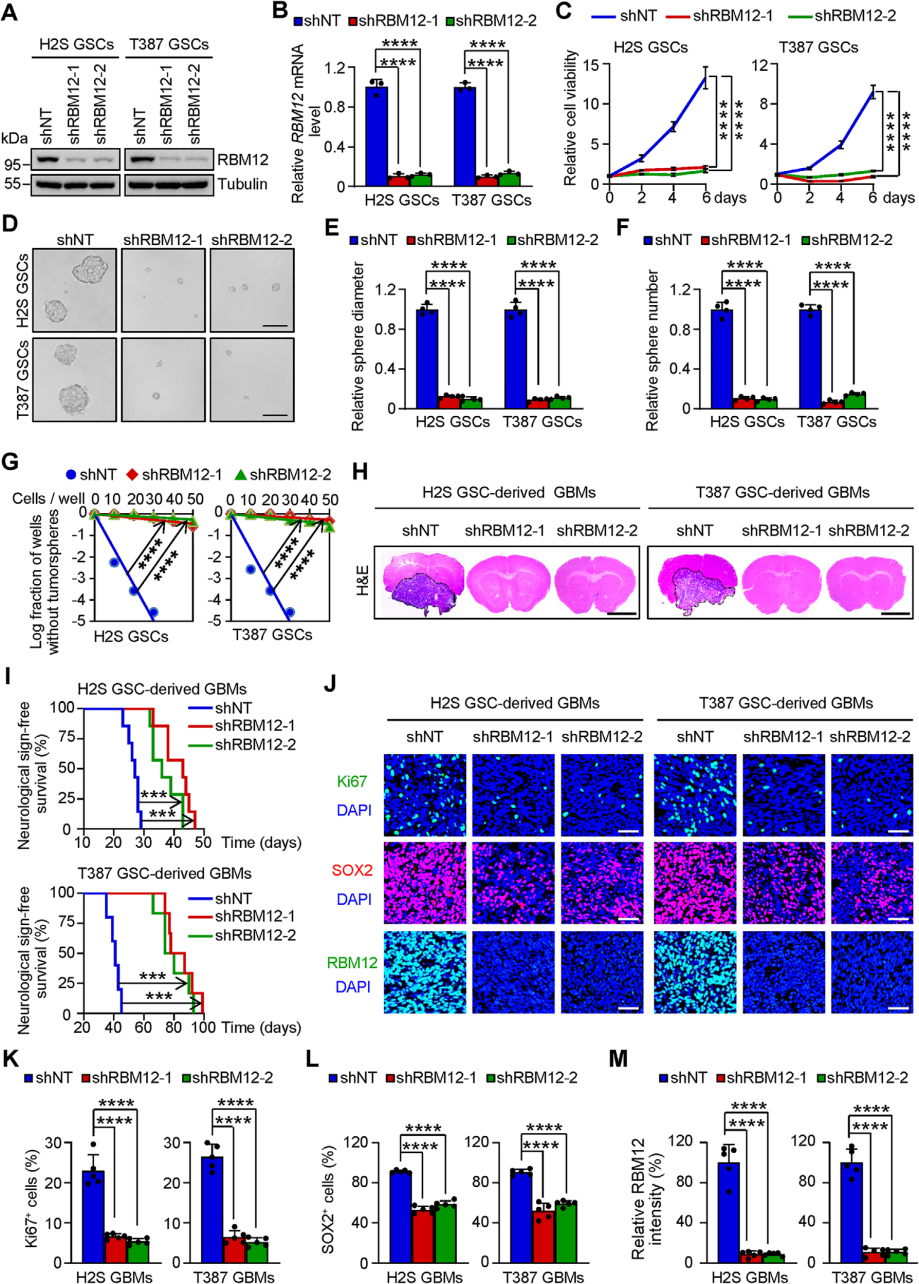
RBM12 is required for GSC proliferation, self‐renewal, and GSC‐driven tumor growth. (A) Immunoblot analysis of RBM12 expression in GSCs transduced with non‐targeting shRNA (shNT) or RBM12 shRNA (shRBM12) through lentiviral infection. (B) qPCR analysis of *RBM12* expression in GSCs transduced with shNT or shRBM12. *n* = 3. (C) Cell viability assay of GSCs transduced with shNT or shRBM12. *n* = 4. (D) Representative images of tumorspheres derived from GSCs expressing shNT or shRBM12. Scale bar, 80 µm. (E, F) Quantification of the diameter (E) and number (F) of tumorspheres derived from GSCs expressing shNT or shRBM12. *n* = 4. (G) In vitro limiting dilution assay of the tumorsphere formations of GSCs expressing shNT or shRBM12. (H) Representative H&E images of mouse brains collected at day 22 (H2S GSCs) or 28 (T387 GSCs) after intracranially transplantation with GSCs expressing shNT or shRBM12. Scale bar, 2 mm. (I) Kaplan–Meier survival curves of mice intracranially transplanted with GSCs expressing shNT or shRBM12. *n* = 5–7 mice per group. (J) Immunofluorescent staining of Ki67 (green), SOX2 (red), or RBM12 (green) in tumor xenografts derived from GSCs expressing shNT or shRBM12. GBM xenografts were collected from mice when neurological signs occurred after GSC transplantation. Scale bar, 50 µm. (K) Quantification of Ki67^+^ cells in tumor xenografts derived from GSCs expressing shNT or shRBM12. *n* = 5 tumors per group. (L) Quantification of SOX2^+^ cells in tumor xenografts derived from GSCs expressing shNT or shRBM12. *n* = 5 tumors per group. (M) Quantification of RBM12 intensity in tumor xenografts derived from GSCs expressing shNT or shRBM12. *n* = 5 tumors per group. Data information: Data are shown as mean ± SD. ^***^ *P* < 0.001, ^****^ *P* < 0.0001, one‐way ANOVA analysis followed by Tukey's test (B, E, F, and K–M), two‐way ANOVA analysis followed by Tukey's test (C), Extreme Limiting Dilution Analysis (ELDA) for differences in stem cell frequencies (G), and log‐rank test (I).

In order to elucidate the role of RBM12 in the maintenance of GSCs, we knocked down RBM12 expression by using two distinct short hairpin RNAs (shRNAs). This targeted knockdown effectively diminished both the protein and mRNA levels of *RBM12* within the GSCs (Figure 2A,B). Subsequent analyses revealed that the suppression of RBM12 led to a significant reduction in cell proliferation, as evidenced by the cell viability and EdU incorporation assays (Figure 2C, Figure S1H,I). However, disrupting RBM12 had minor impact on the growth of NSTCs (Figure S1J,K). Moreover, tumorsphere formation and in vitro limiting dilution assays indicated a pronounced impairment in the self‐renewal capability of GSCs when RBM12 was disrupted (Figure 2D–G). In contrast, the overexpression of RBM12 was found to enhance both cell proliferation and the self‐renewal of GSCs, as depicted in Figure S1L–Q. We next examined RBM12 protein levels across three GSC subtypes (mesenchymal, proneural, and classical) by immunoblot analysis. The results showed that RBM12 expression was largely consistent across all tested subtypes (Figure S2A). Notably, RBM12 disruption also dramatically impaired cell growth in all tested GSCs (Figure S2B–D), indicating that the functional requirement for RBM12 is not restricted to a particular subtype or cellular state. To assess the role of RBM12 in the propagation of GSCs in vivo, we investigated the impact of RBM12 inhibition on GSC‐driven tumor growth. We established orthotopic GBM xenografts by intracranially injecting the GSCs expressing shNT or shRBM12. Hematoxylin and eosin (H&E) staining indicated that disruption of RBM12 in GSCs significantly impeded the growth of intracranial tumors (Figure 2H). As a result, the survival of mice bearing the xenografts derived from GSCs expressing shRBM12 was significantly prolonged compared to that of the control group (Figure 2I). Immunofluorescent staining revealed that silencing RBM12 dramatically reduced the number of Ki67‐positive proliferative cells and the SOX2‐positive GSC population within the tumor xenografts (Figure 2J–M). Collectively, these findings demonstrate that RBM12 plays a pivotal role in promoting the proliferation, self‐renewal and tumorigenic potential of GSCs.

### 2.3 RBM12 Upregulates SLC7A5 to Increase Amino Acid Levels and Activate mTORC1 Signaling in GSCs

**FIGURE 3:**
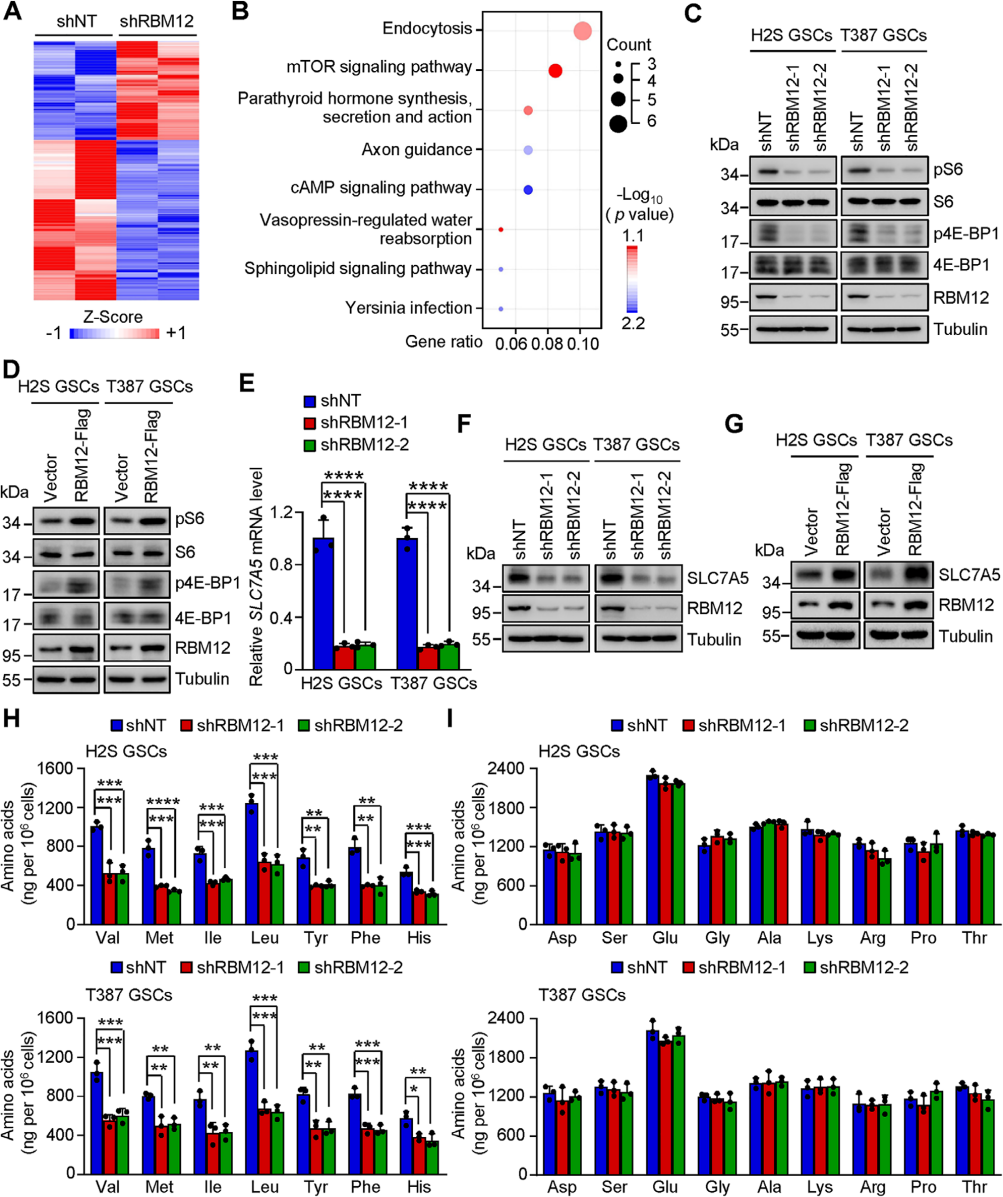
RBM12 promotes SLC7A5 expression to increase amino acid levels and mTORC1 signaling in GSCs. (A) Heatmap analysis reveals the differentially expressed genes between RBM12‐silenced H2S GSCs (H2S) and control H2S GSCs (fold change > 1.8). Among these differentially expressed genes, 100 were upregulated, while 162 were downregulated. (B) KEGG enrichment analysis of downregulated genes in RBM12‐silenced GSCs (H2S). (C) Immunoblot analysis of phospho S6 (pS6), S6, phospho 4E‐BP1 (p4E‐BP1), 4E‐BP1, and RBM12 protein levels in GSCs transduced with shNT or shRBM12. (D) Immunoblot analysis of pS6, S6, p4E‐BP1, 4E‐BP1, and RBM12 protein levels in GSCs transduced with vector control or RBM12‐Flag. (E) qPCR analysis of *SLC7A5* expression in GSCs transduced with shNT or shRBM12. *n* = 3. (F) Immunoblot analysis of SLC7A5 and RBM12 expression in GSCs transduced with shNT or shRBM12. (G) Immunoblot analysis of SLC7A5 and RBM12 expression in GSCs transduced with vector control or RBM12‐Flag. (H) Intracellular levels of amino acids imported via SLC7A5 in GSCs expressing shNT or shRBM12. *n* = 3. (I) Intracellular levels of non‐SLC7A5‐substrate amino acids in GSCs expressing shNT or shRBM12. *n* = 3. Data information: Data are presented as mean ± SD. ^*^ *P* < 0.05, ^**^ *P* < 0.01, ^***^ *P* < 0.001, ^****^ *P* < 0.0001, one‐way ANOVA analysis followed by Tukey's test (E, H, and I).

To uncover the molecular mechanisms by which RBM12 promotes GSC proliferation and self‐renewal, we conducted transcriptional profiling to compare the gene expression of GSCs transduced with shRBM12 to that of those with shNT. GSCs expressing shRBM12 exhibited distinct gene expression profiles relative to control GSCs, with a significant downregulation of 162 genes and upregulation of 100 genes identified in the RBM12‐silenced cells (Figure 3A). Kyoto Encyclopedia of Genes and Genomes (KEGG) enrichment analysis suggested that the downregulated genes were involved in the mTOR signaling pathway (Figure 3B). Therefore, we assessed the expression levels of key markers (phospho S6 and phospho 4E‐BP1) for the mTORC1 pathway in GSCs following RBM12 knockdown or overexpression. Our findings indicated that the phosphorylation levels of S6 and 4E‐BP1 were reduced upon RBM12 knockdown and increased upon RBM12 overexpression (Figure 3C,D). Collectively, these findings underscore the pivotal role of RBM12 in activating mTORC1 signaling. Next, we examined the expression levels of the five genes enriched in the mTOR pathway identified by KEGG analysis (Table S1). PCR analysis revealed that *RHOA*, *SLC7A5*, *STRADB*, and *LRP5* were expressed in GSCs, while *FZD2* was undetectable (Figure S3A). Among these four genes, only *SLC7A5* was significantly higher in GSCs than in NSTCs, whereas *RHOA*, *STRADB*, and *LRP5* showed comparable levels between the two cell populations (Figure S3B–E). SLC7A5 is crucial for the transport of large neutral amino acids into cells, thereby leading to mTORC1 activation and subsequent cell proliferation [28, 29, 30]. These findings prompted us to hypothesize that SLC7A5 might be a key target of RBM12 in promoting mTORC1 pathway activation and GSC proliferation. To test this hypothesis, we examined the impact of RBM12 disruption or overexpression on SLC7A5 expression in GSCs. Consistent with the RNA‐seq result, both mRNA and protein levels of *SLC7A5* were found to be reduced upon RBM12 disruption and increased upon RBM12 overexpression (Figure 3E–G and Figure S3F). Given that SLC7A5 forms a heterodimer with SLC3A2 to function properly, we also investigated the expression of *SLC3A2* in GSCs following RBM12 manipulation. However, our results showed that RBM12 did not influence the expression levels of *SLC3A2* (Figure S3G,H). Subsequently, we aimed to determine whether RBM12 affects the intracellular levels of large neutral amino acids. We found that RBM12 knockdown led to a reduction in the steady‐state levels of large neutral amino acids regulated by SLC7A5 (Figure 3H), while other amino acids remained unchanged (Figure 3I). In conclusion, our data suggest that RBM12 may regulate the intracellular levels of large neutral amino acids and downstream mTORC1 signaling by promoting SLC7A5 expression, thereby facilitating GSC proliferation and self‐renewal.

### 2.4 SLC7A5 Is Also Enriched in GSCs and Is Essential for Their Proliferation and Self‐Renewal

**FIGURE 4:**
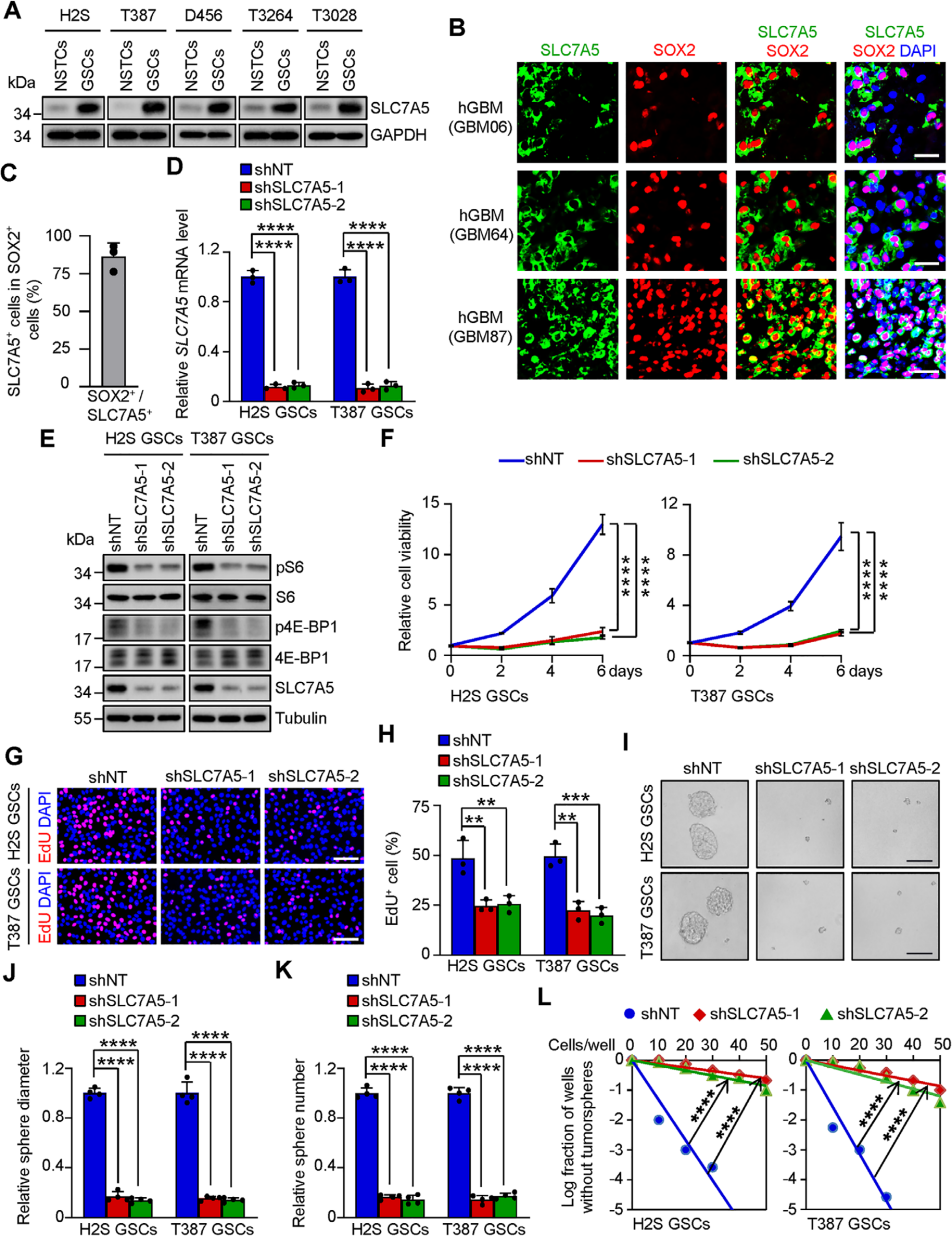
SLC7A5 is essential for GSC growth and self‐renewal. (A) Immunoblot analysis of SLC7A5 expression in five pairs of matched GSCs and NSTCs. (B) Immunofluorescent staining of SLC7A5 (green) and SOX2 (red) in human GBM specimens. Scale bar, 40 µm. (C) Quantification of the fraction of SLC7A5^+^ cells in SOX2^+^ cells in three independent human GBMs. *n* = 3. (D) qPCR analysis of *SLC7A5* expression in GSCs transduced with shNT or shSLC7A5. *n* = 3. (E) Immunoblot analysis of pS6, S6, p4E‐BP1, 4E‐BP1, and SLC7A5 protein levels in GSCs transduced with shNT or shSLC7A5. (F) Cell viability assay of GSCs transduced with shNT or shSLC7A5. *n* = 4. (G) EdU incorporation assay of GSCs transduced with shNT or shSLC7A5. Scale bar: 60 µm. (H) Quantification of the fraction of EdU^+^ cells in GSCs expressing shNT or shSLC7A5. *n* = 3. (I) Representative images of tumorspheres derived from GSCs expressing shNT or shSLC7A5. Scale bar, 80 µm. (J, K) Quantification of the diameter (J) and number (K) of tumorspheres derived from GSCs expressing shNT or shSLC7A5. *n* = 4. (L) In vitro limiting dilution assay of the tumorsphere formations of GSCs expressing shNT or shSLC7A5. Data information: Data are shown as mean ± SD. ^**^ *P* < 0.01, ^***^ *P* < 0.001, ^****^ *P* < 0.0001, one‐way ANOVA analysis followed by Tukey's test (D, H, J, and K), two‐way ANOVA analysis followed by Tukey's test (F), and ELDA analysis for differences in stem cell frequencies (L).

Given that SLC7A5 might be a key target of RBM12 in promoting GSC proliferation and self‐renewal, we proceeded to explore whether SLC7A5 is also essential for their functionality. Immunoblot analyses indicated that SLC7A5 was more abundantly expressed in GSCs relative to matched NSTCs (Figure 4A). Immunofluorescent analysis further confirmed that SLC7A5 was preferentially expressed in glioma cells expressing the GSC marker SOX2 in human GBMs (Figure 4B,C). Immunoblot analysis also showed that the expression levels of SLC7A5 progressively decreased during serum‐induced GSC differentiation (Figure S3I). To elucidate the role of SLC7A5 in GSC maintenance, we knocked down SLC7A5 expression in GSCs using two distinct shRNAs. The knockdown effectively reduced SLC7A5 expression (Figure 4D,E). Moreover, this reduction led to a decrease in the phosphorylation of S6 and 4E‐BP1 in GSCs (Figure 4E). However, disruption of SLC7A5 had no significant effect on the mRNA or protein levels of *RBM12* (Figure S3J,K). Further studies revealed that disruption of SLC7A5 markedly reduced cell proliferation (Figure 4F–H). Additionally, the disruption of SLC7A5 compromised the self‐renewal capacity of GSCs (Figure 4I–L). Taken together, these findings demonstrate that SLC7A5 is also preferentially expressed by GSCs and is indispensable for their proliferation and self‐renewal.

### 2.5 RBM12 Promotes GSC Maintenance and GBM Growth Through SLC7A5‐mTORC1 Pathway

**FIGURE 5:**
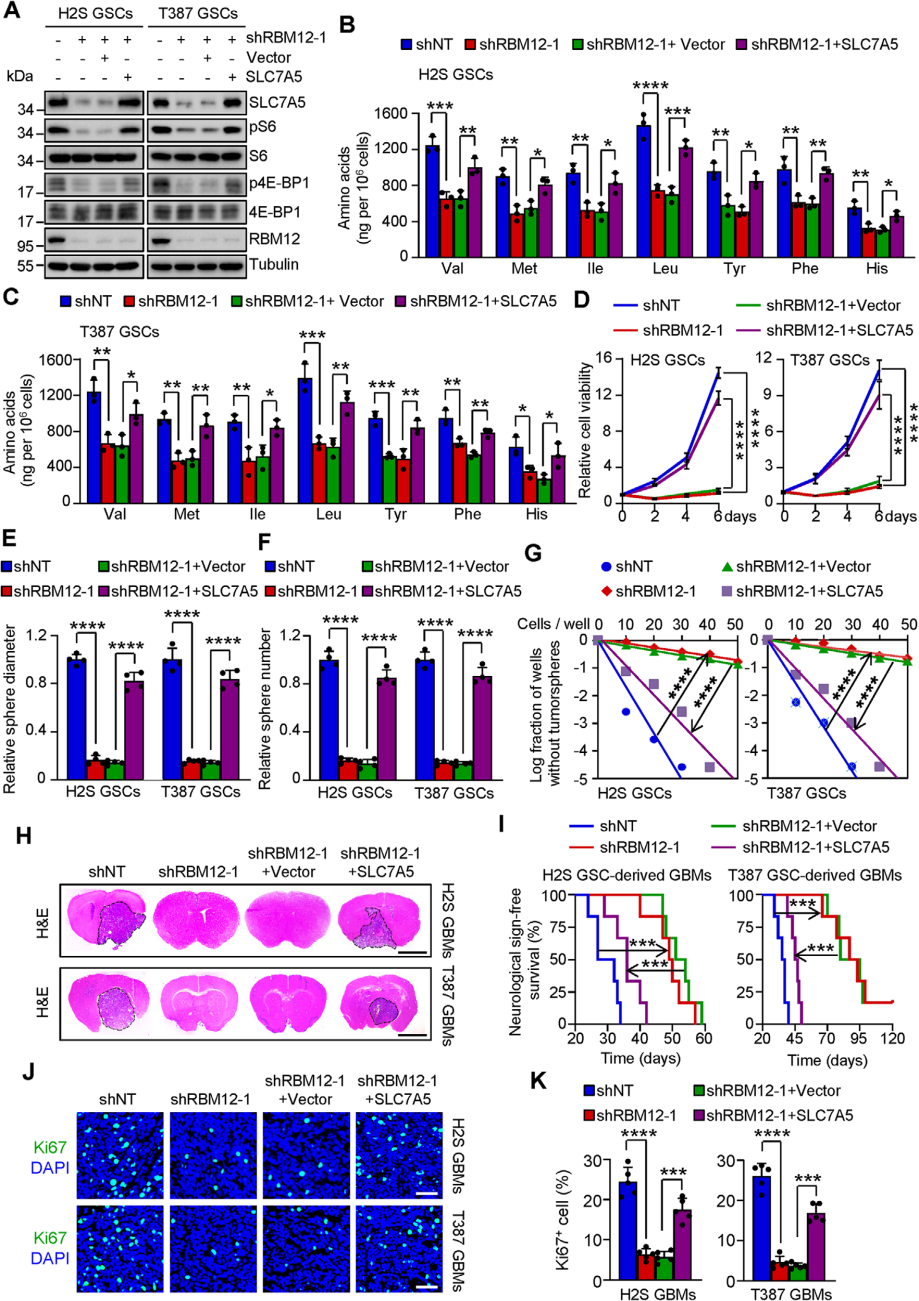
Ectopic expression of SLC7A5 rescues the effects caused by RBM12 disruption. (A) Immunoblot analysis of SLC7A5, pS6, S6, p4E‐BP1, 4E‐BP1, and RBM12 protein levels in GSCs transduced with shNT, shRBM12‐1, shRBM12‐1+Vector, or shRBM12‐1+SLC7A5. (B, C) Intracellular levels of amino acids imported via SLC7A5 in GSCs (H2S, B, and T387, C) expressing shNT, shRBM12‐1, shRBM12‐1+Vector, or shRBM12‐1+SLC7A5. *n* = 3. (D) Cell viability assay of GSCs transduced with shNT, shRBM12‐1, shRBM12‐1+Vector, or shRBM12‐1+SLC7A5. *n* = 4. (E, F) Relative diameter (E) and number (F) of tumorspheres derived from GSCs expressing shNT, shRBM12‐1, shRBM12‐1+Vector, or shRBM12‐1+SLC7A5. *n* = 4. (G) In vitro limiting dilution assay of the tumorsphere formations of GSCs expressing shNT, shRBM12‐1, shRBM12‐1+Vector, or shRBM12‐1+SLC7A5. (H) Representative H&E images of mouse brains collected at day 22 (H2S GSCs) or 28 (T387 GSCs) after intracranially transplantation with GSCs expressing shNT, shRBM12‐1, shRBM12‐1+Vector, or shRBM12‐1+SLC7A5. Scale bar, 2 mm. (I) Kaplan–Meier survival curves of mice intracranially transplanted with GSCs expressing shNT, shRBM12‐1, shRBM12‐1+Vector, or shRBM12‐1+SLC7A5. *n* = 6 mice per group. (J) Immunofluorescent staining of Ki67 (green) in tumor xenografts derived from GSCs expressing shNT, shRBM12‐1, shRBM12‐1+Vector, or shRBM12‐1+SLC7A5. GBM xenografts were collected from mice when neurological signs occurred after GSC transplantation. Scale bar, 50 µm. (K) Quantification of Ki67^+^ cells in tumor xenografts derived from GSCs expressing shNT, shRBM12‐1, shRBM12‐1+Vector, or shRBM12‐1+SLC7A5. *n* = 5 tumors per group. Data information: Data are presented as mean ± SD. ^*^ *P* < 0.05, ^**^ *P* < 0.01, ^***^ *P* < 0.001, ^****^ *P* < 0.0001, one‐way ANOVA analysis followed by Tukey's test (B, C, E, F, and K), two‐way ANOVA analysis followed by Tukey's test (D), ELDA analysis for differences in stem cell frequencies (G), and Log‐rank test (I).

Given that SLC7A5 promotes GSC proliferation and self‐renewal and is regulated by RBM12, we explored whether SLC7A5 mediates the effects of RBM12 on GSC maintenance and GBM growth. To address this question, we examined whether overexpression of SLC7A5 could rescue the impairments caused by RBM12 disruption in GSCs. First, we confirmed that exogenous SLC7A5 could interact with endogenous SLC3A2 in GSCs (Figure S4A). Functionally, overexpression of SLC7A5 rescued the impaired mTORC1 signaling caused by RBM12 knockdown (Figure 5A). Moreover, it restored the reduced levels of large neutral amino acids resulting from RBM12 knockdown (Figure 5B,C, Figure S4B,C). Additionally, overexpression of SLC7A5 rescued the impaired proliferation and self‐renewal of GSCs caused by RBM12 disruption (Figure 5D–G and Figure S4D). Consistently, overexpression of SLC7A5 in GSCs expressing shRBM12 restored GBM tumor growth and attenuated the increased survival of mice bearing the GSC‐derived tumors (Figure 5H,I). Further investigation revealed that overexpression of SLC7A5 rescued the impaired in vivo cell proliferation in xenografts expressing shRBM12, as evidenced by Ki67 immunofluorescence (Figure 5J,K). Furthermore, overexpression of SLC7A5 restored GSC population in xenografts expressing shRBM12, as indicated by SOX2 immunofluorescence (Figure S4E,F). Given that SLC7A5 activates the mTORC1 pathway to promote cell proliferation, we next explored whether RBM12 promotes GSC proliferation and self‐renewal through the mTORC1 pathway. To this end, we transduced GSCs with lentiviral‐mediated overexpression of RBM12 and treated the cells with the mTORC1 inhibitor rapamycin or a vehicle control. The results showed that rapamycin treatment prevented the activation of the mTORC1 pathway induced by RBM12 overexpression (Figure S4G). Rapamycin treatment also inhibited the enhancement of cell proliferation and self‐renewal induced by RBM12 overexpression (Figure S4H,I). In summary, our data demonstrate that RBM12 activates the SLC7A5‐mTORC1 pathway to promote GSC proliferation, self‐renewal and GSC‐driven tumor growth.

### 2.6 RBM12 Stabilizes *SLC7A5* Transcripts in an m^6^A‐Dependent Manner

**FIGURE 6:**
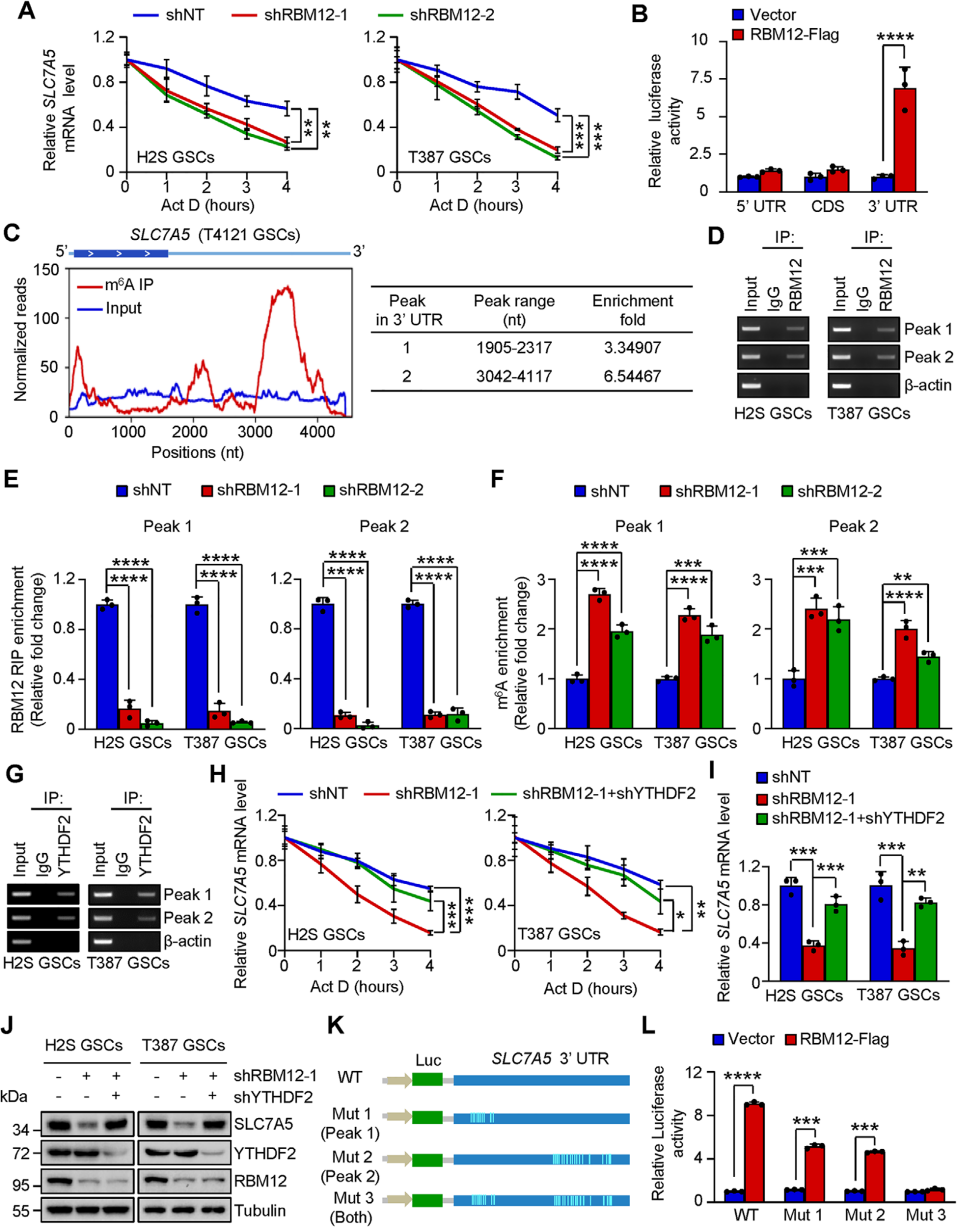
RBM12 promotes SLC7A5 mRNA stability in an m^6^A‐dependent way. (A) GSCs expressing shNT or shRBM12 were treated with 9 µg/mL actinomycin D (ActD) and then harvested at the indicated times for qPCR analysis. *n* = 3. (B) Reporter gene assays were performed in HEK293T cells transfected with *SLC7A5* 5’ UTR, CDS, or 3’ UTR reporter constructs in combination with RBM12‐Flag or vector control. *n* = 3. (C) Left: MeRIP‐seq analysis from the GSE158741 dataset shows the relative abundance of m^6^A sites along the *SLC7A5* mRNA in GSCs (T4121). Right: Identification of m^6^A peaks in the 3’ UTR of *SLC7A5*. (D) RIP‐PCR analysis of RBM12 enrichment at two m^6^A peak regions in the *SLC7A5* 3’ UTR of GSCs. (E) RIP‐qPCR analysis of RBM12 enrichment at two m^6^A peak regions in the *SLC7A5* 3’ UTR of GSCs expressing shNT or shRBM12. *n* = 3. (F) MeRIP‐qPCR analysis of m^6^A enrichment at two m^6^A peak regions in the *SLC7A5* 3’ UTR of GSCs expressing shNT or shRBM12. *n* = 3. (G) RIP‐PCR analysis of YTHDF2 enrichment at two m6A peak regions in the *SLC7A5* 3’ UTR of GSCs. (H) GSCs expressing shNT, shRBM12‐1, or shRBM12‐1+shYTHDF2 were treated with 9 µg/mL ActD and then harvested at the indicated times for qPCR analysis. *n* = 3. (I) qPCR analysis of *SLC7A5* expression in GSCs transduced with shNT, shRBM12‐1, or shRBM12‐1+shYTHDF2. *n* = 3. (J) Immunoblot analysis of SLC7A5, YTHDF2, and RBM12 expression in GSCs transduced with shNT, shRBM12‐1, or shRBM12‐1+shYTHDF2. (K) Schematic diagram of m^6^A site mutations (A to T) in the *SLC7A5* 3’ UTR reporter construct, showing mutations at Peak 1 (Mut 1), Peak 2 (Mut 2), or both regions (Mut 3). (L) Reporter gene assays were performed in HEK293T cells transfected with wild‐type (WT) *SLC7A5* 3’ UTR reporter, *SLC7A5* 3’ UTR Mut 1 reporter, *SLC7A5* 3’ UTR Mut 2 reporter, or *SLC7A5* 3’ UTR Mut 3 reporter, in combination with RBM12‐Flag or vector control. *n* = 3. Data information: Data are shown as mean ± SD. ^*^ *P* < 0.05, ^**^ *P* < 0.01, ^***^ *P* < 0.001, ^****^ *P* < 0.0001, two‐way ANOVA analysis followed by Tukey's test (A and H), two‐tailed unpaired *t*‐test (B and L), and one‐way ANOVA analysis followed by Tukey's test (E, F, and I).

To explore the underlying mechanism by which RBM12 regulates SLC7A5 expression, we first examined whether RBM12 influences the transcription of *SLC7A5*. Our results indicated that overexpression of RBM12 had no effect on *SLC7A5* promoter activity (Figure S5A). In addition, analysis of our RNA‐seq data revealed no splicing changes in *SLC7A5* after RBM12 knockdown (Figure S5B and Table S2). We then assessed the stability and subcellular localization of *SLC7A5* mRNA in GSCs. Interestingly, actinomycin D (ActD) treatment revealed that the stability of *SLC7A5* mRNA was decreased upon RBM12 knockdown and increased upon RBM12 overexpression (Figure 6A and Figure S5C). These results demonstrate that RBM12 regulates the stability of *SLC7A5* mRNA. On the other hand, RBM12 knockdown or overexpression did not appear to affect the nuclear retention or export of *SLC7A5* mRNA (Figure S5D,E). To determine whether RBM12 stabilizes *SLC7A5* mRNA by binding to it, we generated luciferase reporter plasmids containing the 5’ UTR, coding sequence (CDS), or 3’ UTR of *SLC7A5* mRNA and co‐transfected them with the RBM12‐Flag plasmid. The results demonstrated that overexpression of RBM12 selectively increased the luciferase activity of the *SLC7A5* 3’ UTR, while having no significant impact on the 5’ UTR or CDS (Figure 6B). This finding suggests that the *SLC7A5* 3’ UTR plays a crucial role in the regulatory mechanism mediated by RBM12. m^6^A is the most abundant mRNA modification in eukaryotic cells [31, 32], and the *SLC7A5* 3’ UTR undergoes extensive m^6^A modification [33]. Given that m^6^A methylation of the mRNA 3’ UTR plays a crucial role in mRNA stability [34, 35], we attempted to explore whether m^6^A methylation of the *SLC7A5* 3’ UTR is involved in the stability regulation by RBM12. From our analysis of previous m^6^A‐seq data in GSCs [36], we identified three statistically significant m^6^A peaks within the *SLC7A5* mRNA, with two peaks located in the 3’ UTR (Figure 6C and Figure S5F). Using specific primers to detect the two peak regions in the 3’ UTR, we found that RBM12 interacts with these regions of the *SLC7A5* mRNA through RNA immunoprecipitation (RIP)‐PCR analysis (Figure 6D). In addition, this binding was attenuated upon RBM12 knockdown and enhanced upon RBM12 overexpression (Figure 6E and Figure S5G). However, m^6^A levels in these two regions were elevated in GSCs expressing shRBM12 and decreased in GSCs overexpressing RBM12 (Figure 6F and Figure S5H). The YTHDF family is the most extensively studied m^6^A readers, and YTHDF2 is the best‐characterized member, generally expressed at higher levels than YTHDF1 and YTHDF3 in most cell types, and serves as a major decay‐inducing reader [37, 38, 39, 40]. We found that YTHDF2 binds to the two m^6^A peak regions in the 3’ UTR of *SLC7A5* mRNA (Figure 6G). Therefore, we hypothesized that YTHDF2 mediates the decay of *SLC7A5* mRNA upon RBM12 knockdown. We demonstrated that combined knockdown of the m^6^A reader YTHDF2 and RBM12 reversed the destabilization and downregulation of *SLC7A5* mRNA resulting from RBM12 single knockdown (Figure 6H,I). Combined knockdown of YTHDF2 and RBM12 also reversed the downregulation of SLC7A5 protein levels caused by RBM12 single knockdown (Figure 6J).

To identify the specific m^6^A sites in *SLC7A5* mRNA, the SRAMP tool was employed to predict m^6^A sites [41]. A total of 29 m^6^A sites with very high confidence were predicted, all of which were located in the 3’ UTR region (Table S3). Among these 29 m^6^A sites, 10 were found in the Peak 1 region, while 18 were found in the Peak 2 region of the *SLC7A5* mRNA 3’ UTR in GSCs (Table S3). To determine whether RBM12‐mediated regulation of the *SLC7A5* mRNA 3’ UTR depends on m^6^A modifications within the two identified m^6^A peak regions of the 3’ UTR, we mutated either the 10 m^6^A sites in the Peak 1 region, the 18 m^6^A sites in the Peak 2 region, or both, and then performed a luciferase reporter assay (Figure 6K). Our results showed that mutating the m^6^A sites in either of the two peak regions significantly reduced the luciferase activity of the 3’ UTR of *SLC7A5* mRNA induced by RBM12 (Figure 6L). In contrast, simultaneous mutation of both regions completely eliminates this induced effect (Figure 6L). These data indicate that RBM12 maintains the stability of *SLC7A5* mRNA by reducing its m^6^A modifications.

### 2.7 RBM12 Recruits ALKBH5 to Stabilize *SLC7A5* Transcripts

**FIGURE 7:**
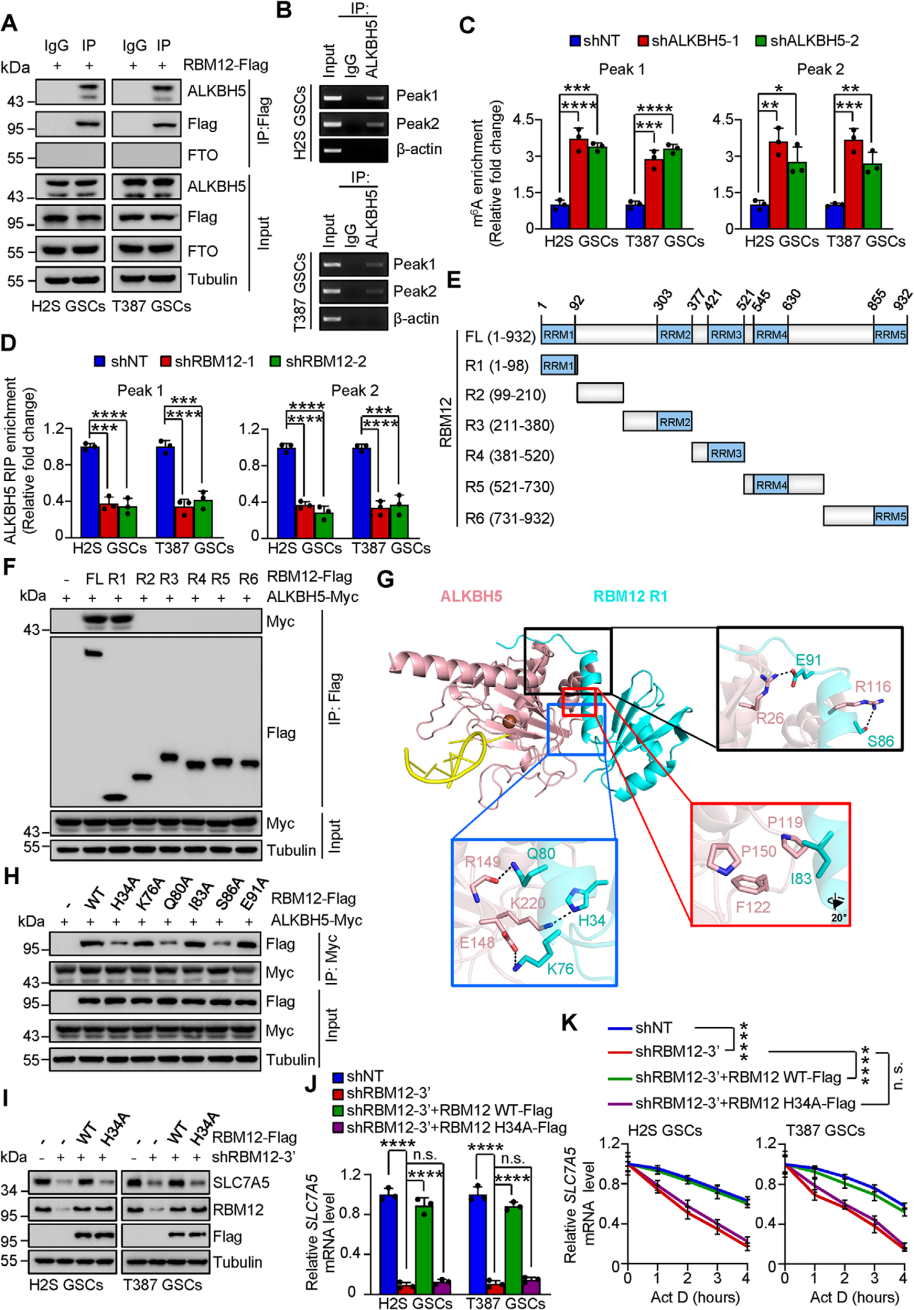
RBM12 cooperates with ALKBH5 to stabilize the SLC7A5 transcripts. (A) CoIP assays of interaction between RBM12‐Flag and the indicated proteins in GSCs expressing RBM12‐Flag. The precipitated proteins and total lysates were then analyzed by immunoblotting using the indicated antibodies. (B) RIP‐PCR analysis of ALKBH5 enrichment at two m^6^A peak regions in the *SLC7A5* 3’ UTR of GSCs. (C) MeRIP‐qPCR analysis of m^6^A enrichment at two m^6^A peak regions in the *SLC7A5* 3’ UTR of GSCs expressing shNT or shALKBH5. *n* = 3. (D) RIP‐qPCR analysis of ALKBH5 enrichment at two m^6^A peak regions in the *SLC7A5* 3’ UTR of GSCs expressing shNT or shRBM12. *n* = 3. (E) Schematic diagram of Flag‐tagged full‐length (FL) RBM12 and its truncated mutants. (F) HEK293T cells were co‐transfected with ALKBH5‐Myc and Flag‐tagged FL RBM12 or its truncated mutants, and cell lysates were analyzed by IP with Flag antibody followed by immunoblotting with indicated antibodies. (G) Overview of the ALKBH5‐RBM12 R1 complex predicted using AlphaFold3. ALKBH5 and RBM12 R1 are shown in pink and cyan, respectively. Close‐up views of selected interaction interface (boxed in black, red, and blue) highlight key residues. Interacting residues from both proteins are represented as stick models, with ALKBH5 and RBM12 R1 residues labeled in their respective colors. (H) HEK293T cells were co‐transfected with ALKBH5‐Myc and Flag‐tagged wild‐type (WT) RBM12 or its site‐specific mutant, and cell lysates were analyzed by IP with a Myc antibody followed by immunoblotting with indicated antibodies. (I) Immunoblot analysis of SLC7A5, RBM12, and Flag expression in GSCs transduced with shNT, shRBM12‐3’ UTR (shRBM12‐3’), shRBM12‐3’+RBM12 WT‐Flag, or shRBM12‐3’+RBM12 H34A‐Flag. (J) qPCR analysis of *SLC7A5* expression in GSCs transduced with shNT, shRBM12‐3’, shRBM12‐3’+RBM12 WT‐Flag, or shRBM12‐3’+RBM12 H34A‐Flag. *n* = 3. (K) GSCs expressing shNT, shRBM12‐3’, shRBM12‐3’+RBM12 WT‐Flag, or shRBM12‐3’+RBM12 H34A‐Flag were treated with 9 µg/mL ActD and then harvested at the indicated times for qPCR analysis. *n* = 3. Data information: Data are presented as mean ± SD. ^*^ *P* < 0.05, ^**^ *P* < 0.01, ^***^ *P* < 0.001, ^****^ *P* < 0.0001, one‐way ANOVA analysis followed by Tukey's test (C, D, and J) and two‐way ANOVA analysis followed by Tukey's test (K). n.s., not significant.

To further elucidate the molecular mechanism by which RBM12 removes m^6^A modifications from *SLC7A5* mRNA, we examined whether RBM12 could recruit an m^6^A demethylase to specifically target and remove these modifications on *SLC7A5* mRNA. We performed a co‐immunoprecipitation (CoIP) experiment in GSCs expressing RBM12‐Flag and found that RBM12 specifically interacts with the endogenous m^6^A demethylase ALKBH5, but not with FTO, another key m^6^A eraser (Figure 7A). Additionally, CoIP assays confirmed that exogenous ALKBH5 was readily detected in exogenous RBM12 pull‐down complexes and vice versa (Figure S6A). We next explored whether ALKBH5 regulates the mRNA stability of *SLC7A5* through binding to its 3’ UTR, similar to RBM12. Our analyses revealed that silencing ALKBH5 significantly reduced the mRNA abundance, protein expression, and mRNA stability of *SLC7A5* in GSCs (Figure S6B–D). However, silencing SLC7A5 had no effect on the mRNA or protein levels of *ALKBH5* (Figure S3J,K). Moreover, we confirmed that ALKBH5 binds to the two m^6^A peak regions in the 3’ UTR of *SLC7A5* (Figure 7B), and this binding was diminished following ALKBH5 knockdown (Figure S6E). Consistent with these findings, m^6^A levels in these two regions were also elevated in GSCs expressing shALKBH5 (Figure 7C). Further analysis demonstrated that the binding of ALKBH5 to these two regions was attenuated upon RBM12 knockdown and enhanced upon RBM12 overexpression (Figure 7D and Figure S6F). Subsequently, we examined the function of ALKBH5 in the maintenance of GSCs. Immunoblot analysis showed that the expression levels of ALKBH5 protein were higher in GSCs than in NSTCs (Figure S7A). In addition, the expression levels of ALKBH5 gradually decreased during serum‐induced GSC differentiation (Figure S3I). Cell viability and EdU incorporation assays demonstrated that disruption of ALKBH5 significantly reduced GSC proliferation (Figure S7B–D). Disruption of ALKBH5 also impaired the self‐renewal capacity of GSCs (Figure S7E–H). As expected, disruption of ALKBH5 led to a decrease in the phosphorylation of S6 and 4E‐BP1 (Figure S7I). Next, we examined whether ALKBH5‐mediated regulation of SLC7A5 protein levels, mRNA abundance, and mRNA stability is dependent on RBM12. Immunoblot and qPCR analyses demonstrated that ALKBH5 overexpression failed to restore the decreased SLC7A5 protein levels, mRNA abundance, and mRNA stability caused by RBM12 knockdown (Figure S7J–L).

To investigate whether the m^6^A demethylase activity of ALKBH5 is required for *SLC7A5* stabilization, we examined whether ectopic expression of wild‐type (WT) or catalytically inactive mutant (H204A) of ALKBH5 could rescue the effects caused by ALKBH5 disruption. We employed an additional shRNA that specifically targets the 3’ UTR of the endogenous *ALKBH5* mRNA (designated as shALKBH5‐3') to knock down ALKBH5 expression. This approach enabled us to simultaneously silence endogenous ALKBH5 and overexpress exogenous ALKBH5 in GSCs. Immunoblot and qPCR analyses revealed that WT ALKBH5, but not the H204A mutant, could rescue the decreased protein levels, mRNA abundance, and mRNA stability of *SLC7A5* caused by knockdown of endogenous ALKBH5 (Figure S8A–C). Additionally, methylated RNA immunoprecipitation (MeRIP)‐qPCR analysis demonstrated that WT ALKBH5, but not the H204A mutant, prevented the increased m^6^A levels in the two m^6^A peak regions of the *SLC7A5* 3’ UTR (Figure S8D).

To explore the specific region of the RBM12 protein required for RBM12‐ALKBH5 interaction, we generated various truncated mutants of RBM12 (Figure 7E). CoIP assay demonstrated that the N‐terminal domain of RBM12 (R1 domain, amino acids 1–98) is required for interaction with ALKBH5 (Figure 7F). To further determine how the RBM12 R1 domain interacts with ALKBH5, we used AlphaFold 3 to predict their interaction [42]. As shown in Figure 7G, the predicted interaction surface between the RBM12 R1 domain and ALKBH5 involves specific amino acids in RBM12, including H34, K76, Q80, I83, S86, and E91. By performing site‐directed mutagenesis followed by CoIP analysis, we found that mutation of the H34, Q80, or S86 residue in RBM12 significantly disrupted the interaction between RBM12 and ALKBH5 (Figure 7H), suggesting that these amino acids in RBM12 are indispensable for the interaction with ALKBH5. Our results also found that these mutations did not alter RBM12 protein expression (Figure S9A), stability (Figure S9B–E) or its subcellular localization (Figure S9F,G). Subsequently, we investigated whether the interaction between RBM12 and ALKBH5 is essential for *SLC7A5* stabilization. We examined whether ectopic expression of WT RBM12, H34A, Q80A, or S86A could rescue the effects caused by RBM12 knockdown. We also employed an additional shRNA that specifically targets the 3’ UTR of the endogenous *RBM12* mRNA (designated as shRBM12‐3') to knock down RBM12 expression. Immunoblot and qPCR analyses revealed that WT RBM12, but not the H34A, Q80A, or S86A mutants, could rescue the decreased protein levels, mRNA abundance, and mRNA stability of *SLC7A5* caused by knockdown of endogenous RBM12 (Figure 7I–K and Figure S10A–C). Moreover, RIP‐qPCR analysis demonstrated that WT RBM12, but not the H34A mutant, rescued the decreased binding of ALKBH5 to the two m^6^A peak regions of the *SLC7A5* 3’ UTR (Figure S10D). Furthermore, MeRIP‐qPCR analysis revealed that WT RBM12, but not the H34A mutant, prevented the increased m^6^A levels in the two m^6^A peak regions of the *SLC7A5* 3’ UTR (Figure S10E). Collectively, these results demonstrate that RBM12 recruits ALKBH5 to remove m^6^A modifications on *SLC7A5* mRNA and maintain its stability.

### 2.8 *RBM12* is Transcriptionally Upregulated by OTX1 in GSCs

To explore the mechanism underlying the increased *RBM12* transcription in GSCs, we analyzed the *RBM12* promoter region (‐2000 to +100) using the JASPAR database to predict transcription factors that regulate *RBM12* transcription. The top 20 transcription factors with the highest scores, along with their potential binding motifs, are listed in Table S4. By integrating these top 20 transcription factors with transcriptome data (GEO datasets GSE247418 and GSE54791) from GSCs and differentiated glioma cells (DGCs), we identified three candidate transcription factors—OTX1, NFATC2, and ZBTB12—that are preferentially expressed in GSCs (Figure S11A). We then examined the mRNA levels of these three factors in GSCs and found that the expression of *OTX1* was much higher than that of *NFATC2* and *ZBTB12* (Figure S11B). Therefore, we verified the preferential expression of OTX1 in GSCs and examined whether OTX1 regulates *RBM12* transcription. Immunoblot and qPCR analyses confirmed that the protein and mRNA expression of *OTX1* are preferentially expressed in GSCs compared with NSTCs (Figure S11C,D). Immunofluorescent analysis further confirmed that OTX1 was preferentially expressed in glioma cells expressing the GSC marker SOX2 in human GBMs (Figure S11E,F). We next silenced OTX1 expression by two independent shRNAs and found that disruption of OTX1 inhibited the protein and mRNA expression of *RBM12* (Figure S11G,H). To determine whether OTX1 directly stimulates the transcription of *RBM12*, we conducted a luciferase reporter assay. The results showed that overexpression of OTX1 increased *RBM12* promoter activity (Figure S11I,J). Mutating either of the two potential OTX1‐binding sites within the *RBM12* promoter attenuated the activating effects of OTX1 (Figure S11I,J). However, the activation was completely abolished when both binding sites were mutated (Figure S11I,J). Chromatin immunoprecipitation (ChIP) assay demonstrated that OTX1 binds to these two sites on the proximal *RBM12* promoter in GSCs expressing OTX1‐Flag (Figure S11K). Taken together, these data indicate that OTX1 promotes *RBM12* transcription by binding to its promoter region in GSCs.

### 2.9 Pharmacologic Inhibition of the RBM12‐SLC7A5 Axis by the SLC7A5 Inhibitor JPH203 Suppresses GSC‐Driven Tumor Growth

**FIGURE 8:**
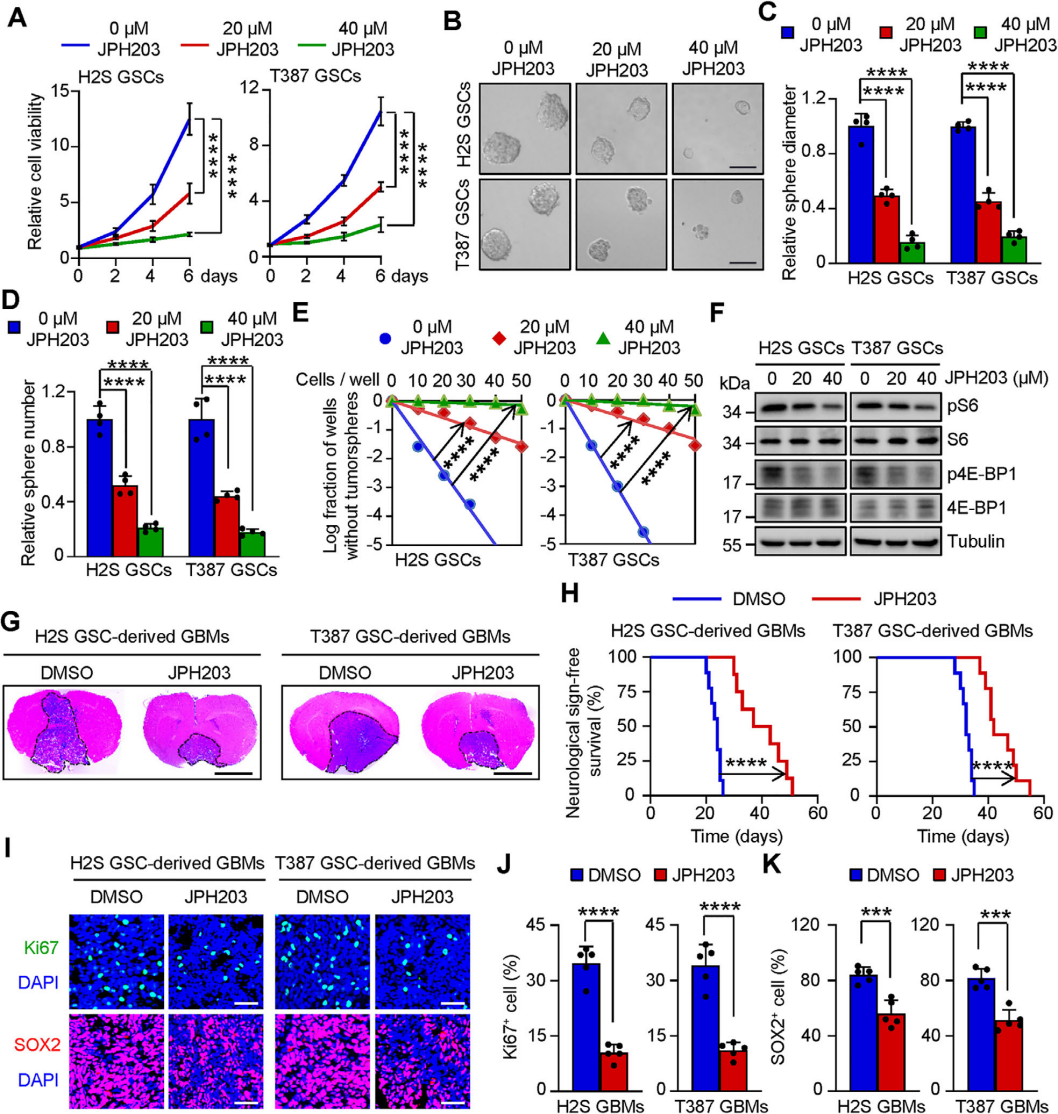
JPH203 treatment inhibited GSC proliferation, self‐renewal, and GSC‐driven tumor growth. (A) Cell viability assay of GSCs treated with the indicated doses of JPH203 or the vehicle control. *n* = 4. (B) Tumorsphere images of GSCs treated with the indicated doses of JPH203 or the vehicle control. Scale bar, 80 µm. (C, D) Quantification of the diameter (C) and number (D) of tumorspheres from GSCs treated with the indicated doses of JPH203 or the vehicle control. *n* = 4. (E) In vitro limiting dilution assay of the tumorsphere formations of GSCs treated with the indicated doses of JPH203 or the vehicle control. (F) Immunoblot analysis of pS6, S6, p4E‐BP1, and 4E‐BP1 protein levels in GSCs treated with the indicated doses of JPH203 or the vehicle control. (G) Representative H&E images of mouse brains collected at day 22 (H2S GSCs) or 28 (T387 GSCs) after intracranially transplantation with GSCs. Seven days post‐GSC transplantation, mice were treated with JPH203 or vehicle control for 6 days per week and maintained until the indicated collection time. Scale bar, 2 mm. (H) Kaplan–Meier survival curves of mice bearing GSC‐derived xenografts treated with JPH203 or vehicle control. Seven days post‐GSC transplantation, mice were treated with JPH203 or vehicle control for 6 days per week and maintained until neurological signs appeared. *n* = 8–9 mice per group. (I) Immunofluorescent staining of Ki67 (green) or SOX2 (red) in GSC‐derived xenografts from mice treated with JPH203 or the vehicle control. GBM xenografts were collected from mice when neurological signs occurred after GSC transplantation. Scale bar, 50 µm. (J) Quantification of Ki67^+^ cells in GSC‐derived xenografts from mice treated with JPH203 or the vehicle control. n = 5 tumors per group. (K) Quantification of SOX2^+^ cells in GSC‐derived xenografts from mice treated with JPH203 or the vehicle control. *n* = 5 tumors per group. Data information: Data are shown as mean ± SD. ^***^ *P* < 0.001, ^****^ *P* < 0.0001, two‐way ANOVA analysis followed by Tukey's test (A), one‐way ANOVA analysis followed by Tukey's test (C and D), ELDA analysis for differences in stem cell frequencies (E), Log‐rank test (H), and two‐tailed unpaired *t*‐test (J, K).

As the RBM12‐SLC7A5 axis is critical for GSC maintenance and no RBM12 inhibitors are available, we reasoned that pharmacologically targeting SLC7A5 with JPH203, a small‐molecule inhibitor, could represent a viable therapeutic strategy to compromise GSCs and suppress GSC‐driven tumor growth. Our results demonstrated that JPH203 treatment significantly inhibited GSC proliferation in a dose‐dependent manner (Figure 8A). Notably, JPH203 displayed preferential activity against GSCs over NSTCs (Figure S12A). Consistently, JPH203 also dose‐dependently impaired GSC self‐renewal (Figure 8B–E). Moreover, JPH203 treatment dose‐dependently suppressed the phosphorylation of S6 and 4E‐BP1 (Figure 8F). However, JPH203 did not induce apoptosis in GSCs, as evidenced by unchanged levels of Cleaved PARP and Cleaved Caspase‐7 (Figure S12B). JPH203 also had no significant effect on RBM12 expression in GSCs (Figure S12B). Subsequently, we assessed the therapeutic impact of JPH203 on the growth of orthotopic GBM xenografts. H&E staining revealed that JPH203 treatment markedly suppressed the growth of GSC‐driven tumors (Figure 8G). Consequently, mice treated with JPH203 exhibited extended survival compared to control mice (Figure 8H). Our study further demonstrated that JPH203 is well‐tolerated, as no significant changes in body weight were observed following the treatment (Figure S12C,D). Additionally, immunofluorescent staining showed that JPH203 administration reduced the number of Ki67‐positive proliferative cells and the SOX2‐positive GSC population within the xenografts (Figure 8I–K). Collectively, these data indicate that the inhibition of the RBM12‐SLC7A5 axis by the SLC7A5 inhibitor JPH203 effectively disrupts GSC maintenance and mitigates GBM growth.

## 3 Discussion

**FIGURE 9:**
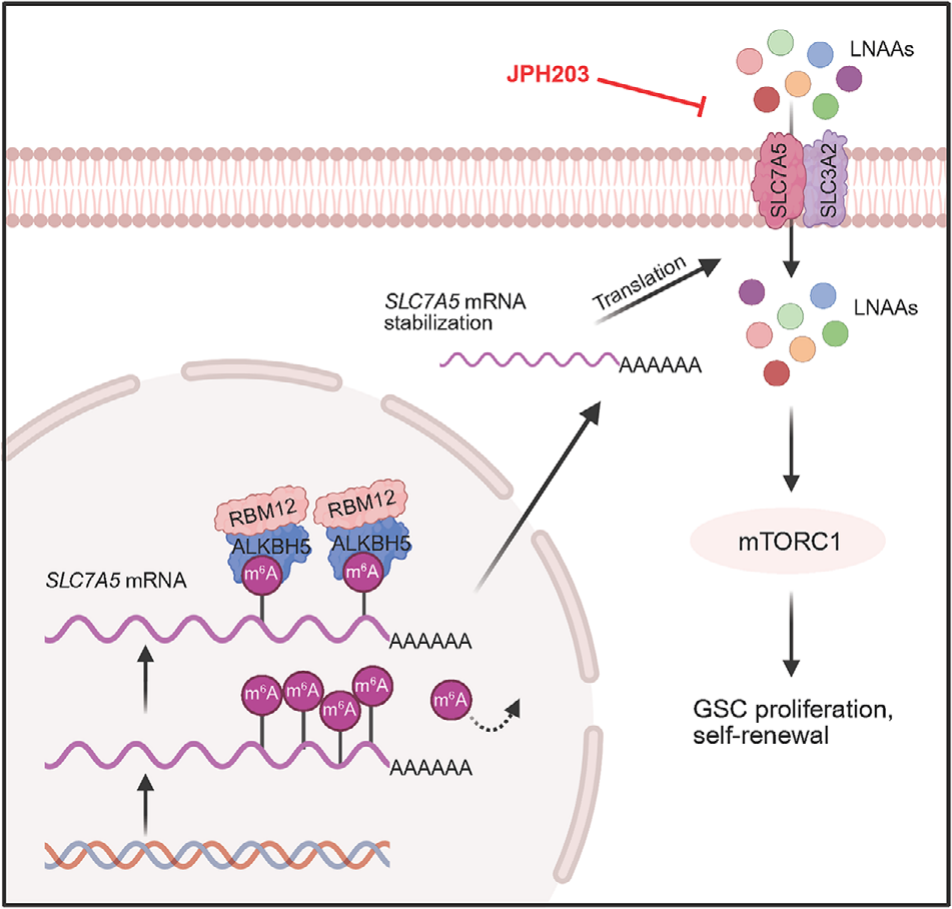
A schematic model illustrating that the RBM12‐ALKBH5 complex promotes GSC proliferation and self‐renewal by stabilizing *SLC7A5* transcripts via m^6^A demethylation, thereby activating amino acid‐dependent mTORC1 signaling. RBM12 enhances *SLC7A5* mRNA stability by recruiting ALKBH5 to remove m^6^A modifications on *SLC7A5* transcripts. This mechanism elevates intracellular large neutral amino acid (LNAA) levels and activates downstream mTORC1 signaling, ultimately promoting GSC proliferation, self‐renewal, and tumor growth. Pharmacological inhibition of this axis using the SLC7A5 inhibitor JPH203 suppresses GSC maintenance and tumor progression. Created in BioRender. Lei, H. (2026) https://BioRender.com/geapgqj.

RBM12 has been shown to be upregulated in hepatocellular carcinoma and to drive the progression of liver cancer [26, 27]. However, the role of RBM12 in the development of other tumors remains unclear. In this study, we found that RBM12 is preferentially expressed in GSCs and is essential for GSC proliferation, self‐renewal, and GSC‐driven tumor growth. We demonstrated that RBM12 promotes GSC maintenance by stabilizing *SLC7A5* mRNA, thereby activating amino acid‐dependent mTORC1 signaling. Moreover, we showed that RBM12 enhances *SLC7A5* mRNA stability by recruiting ALKBH5 to remove m^6^A modifications on *SLC7A5* mRNA. Pharmacological inhibition of the RBM12‐SLC7A5 axis using the SLC7A5 inhibitor JPH203 significantly suppressed GSC proliferation, self‐renewal, and GBM tumor growth (Figure 9). Therefore, targeting the RBM12‐SLC7A5 signaling pathway may provide the potential to improve the efficacy of GBM treatment.

As RBM12 is enriched in GSCs, we utilized the JASPAR database to predict transcription factors that regulate *RBM12* transcription. Through this prediction and subsequent experimental validation, we identified OTX1 as an upstream transcription factor that promotes *RBM12* transcription. OTX1 is a homeobox‐containing transcription factor that is essential for brain morphogenesis [43]. Accumulating studies have demonstrated that OTX1 promotes tumor progression in various types of cancer, including ovarian, cervical, colorectal, and liver cancers, among others [44, 45, 46, 47, 48]. Our results demonstrated that OTX1 enhances RBM12 expression in GSCs, suggesting that OTX1 may also function as an oncogene that promotes GBM progression. Future studies are needed to confirm whether OTX1 indeed promotes GSC maintenance and GSC‐driven tumor growth. Additionally, it remains unclear whether OTX1 is regulated by RBM12 or ALKBH5 in GSCs.

Cancer cells require an increased influx of amino acids to sustain their elevated protein synthesis and cell proliferation [49]. Specialized transporters mediate the exchange of amino acids across cell membranes [50]. SLC7A5, a key member of these transporters, exports glutamine out of cells while importing large neutral amino acids into cells, thereby activating mTORC1 [28, 51]. Among these amino acids, leucine is the best‐established, and its deprivation strongly inhibits mTORC1 [52, 53]. SLC7A5 is often overexpressed in cancer cells, thus promoting cell proliferation [54]. However, the precise regulatory mechanisms underlying aberrant SLC7A5 expression remain incompletely understood. Our study identified RBM12 as a critical upstream regulator of SLC7A5 in GSCs. We further established that RBM12 binds to ALKBH5 and stabilizes *SLC7A5* mRNA via an m^6^A‐dependent mechanism, revealing a post‐transcriptional regulatory pathway. A recent study reported that RBM12 promotes the translation of *JAK1* mRNA, thereby driving liver cancer progression [27]. In that context, RBM12 was detected in both the cytoplasm and nucleus of hepatoma cells [27]. In contrast, we found that RBM12 is predominantly localized in the nucleus of GSCs, suggesting that its regulatory mechanisms may depend on subcellular localization. Previous work showed that ALKBH5 reduces m^6^A modifications on *SLC7A5* mRNA in human urothelial cancer cells, leading to decreased SLC7A5 protein levels without affecting its mRNA abundance [55]. The authors further demonstrated that ALKBH5 inhibits *SLC7A5* translation [55]. However, our experiments revealed that in GSCs, ALKBH5‐mediated reduction of m^6^A modifications on *SLC7A5* mRNA results in decreased mRNA stability. These findings suggest that the regulatory mechanism of ALKBH5 on SLC7A5 may be cell type‐dependent. Moreover, pharmacological inhibition of the RBM12‐SLC7A5 axis using the SLC7A5 inhibitor JPH203 markedly impaired GSC maintenance and tumor growth, suggesting that targeting this axis may be an effective therapeutic strategy against GBM. Based on published binding specificity [54, 56, 57], JPH203's suppression of mTORC1 and GSC maintenance is likely mediated via SLC7A5 inhibition, reinforced by the observation that genetic knockdown of SLC7A5 phenocopies JPH203's effects, supporting an on‐target mechanism. Recently, a phase I clinical trial demonstrated the safety and efficacy of JPH203 in patients with advanced solid tumors [58]. Thus, JPH203 may represent a promising candidate for improving GBM therapy. Future studies should aim to identify RBM12‐specific inhibitors and evaluate whether targeting RBM12 could enhance therapeutic outcomes in GBM.

In summary, our study identifies the RBM12/ALKBH5 complex as a crucial regulator that activates the amino acid‐dependent mTORC1 pathway via SLC7A5, thereby promoting the maintenance of GSCs and the growth of GBM. The RBM12/ALKBH5 complex enhances the stability of *SLC7A5* mRNA by binding to its 3’ UTR and removing m^6^A modifications in this region. Given that pharmacological inhibition of SLC7A5 significantly suppresses GSC maintenance and GBM growth, targeting the RBM12/ALKBH5‐SLC7A5 axis may enhance therapeutic outcomes for GBM patients.

## 4 Experimental Section

### 4.1 GBM Specimens

Human GBM specimens were collected from GBM patients at TaiHe Hospital (The Affiliated Hospital of Hubei University of Medicine, Shiyan, China) in accordance with an Institutional Research Ethics Committee‐approved protocol. Informed consent was obtained from all patients. All procedures involving human tissues were conducted in accordance with the principles of the Declaration of Helsinki and were approved by the Ethics Committee of Taihe Hospital (Approval No. 2023KS11).

### 4.2 Cell Culture

GSCs and NSTCs were generously provided by Dr. Jeremy N. Rich (University of North Carolina at Chapel Hill, USA) and Dr. Shideng Bao (Cleveland Clinic, USA). These cells were isolated from human GBM specimens or patient‐derived GBM xenografts and functionally characterized [59, 60, 61]. Briefly, the cells were dissociated using the Papain Dissociation System (Worthington Biochemical, Cat# LK003150) according to the manufacturer's instructions. The dissociated tumor cells were recovered for at least 6 h in the Neurobasal‐A medium (Shanghai BasalMedia, Cat# T721KJ), supplemented with B27 (Thermo Fisher, Cat# 12587010), 2mm L‐glutamine (Shanghai BasalMedia, Cat# S240JV), 1mm sodium pyruvate (Shanghai BasalMedia, Cat# S410JV), 20 ng/mL bFGF (PrimeGene, Cat# 104‐02), and 20 ng/mL EGF (Gold biotechnology, Cat# 1150‐04‐1000). The isolated cells were then sorted using the PE‐conjugated anti‐CD133 antibody (Miltenyi Biotec, Cat# 130‐113‐108) and FITC‐conjugated anti‐CD15 antibody (BD Biosciences, Cat# 347423) to obtain the GSCs (CD133^+^/CD15^+^) and NSTCs (CD133^−^/CD15^−^). The cancer stem cell phenotypes of the isolated GSCs were validated by the expression of GSC markers (SOX2, OLIG2, CD133, and L1CAM) and a series of functional assays, including tumorsphere formation, serum‐induced cell differentiation, in vivo tumor formation, and in vivo limiting dilution assays. NSTCs were maintained in DMEM with 10% FBS (Zhejiang TianHang Biotech, Cat# 70220–8611) to maintain their differentiation status. ENStem‐A NPCs (Millipore, Cat # SCC003), hNP1 NPCs (Neuromics, Cat # HN60001), and 15167 NPCs (kindly provided by Dr. Shideng Bao) were also cultured in Neurobasal‐A medium supplemented with the same components as used for GSCs.

### 4.3 RNA Extraction and mRNA Analysis

For cellular total RNA extraction, the procedure was performed using the TRIzol reagent (Takara, Cat# 9109) according to the manufacturer's protocol. For the separation of nuclear and cytoplasmic fractions, the extraction kit (Biosharp, Cat# BL670A) was used. The RNA concentration was measured using the Nanodrop 2000c spectrophotometer (Thermo Fisher). Subsequently, the RNA was reverse transcribed into cDNA using PrimeScript RT Master Mix (Takara, Cat# RR036A). PCR was carried out using Taq Master Mix (Vazyme, Cat# P111‐01) on a T100 Thermal Cycler (Bio‐Rad). For quantitative real‐time PCR (qPCR), it was performed using Taq Pro Universal SYBR qPCR Master Mix (Vazyme, Cat# Q712‐02) on a 7500 Fast Real‐Time PCR System (Applied Biosystems). Sequences of PCR/qPCR primers were listed in Table S5.

### 4.4 RNA‐seq and Data Analysis

GSCs were transduced with shNT or shRBM12. Total RNA was isolated using the TRIzol reagent (Takara, Cat# 9109) according to the manufacturer's instructions. The extracted RNA was then subjected to RNA‐seq analysis. A small portion of each RNA sample was analyzed by qPCR to assess knockdown efficiency prior to RNA‐seq. Library construction and sequencing were performed using the Illumina platform at Novogene. KEGG analysis was conducted using the Sangerbox 3.0 tool.

### 4.5 Plasmid Construction, Site‐Directed Mutagenesis and Lentiviral Production

Lentiviral shRNAs targeting human *RBM12*, *SLC7A5*, *ALKBH5*, *YTHDF2*, *OTX1*, and a non‐targeting shRNA were cloned into pLKO.1‐Puro (Addgene, Cat# 8453) or pLKO.1‐Hygro (MingLingBio, Cat# P0702) vectors. The shRNA sequences were listed in Table S5. Human *RBM12‐Flag*, *SLC7A5*, *SLC7A5‐Flag*, *ALKBH5‐Myc*, and *OTX1‐Flag* were generated by cloning their coding sequences into the pLVX‐EF1α‐IRES‐Puro (MingLingBio, Cat# P0405) or pLVX‐EF1α‐IRES‐Neo vectors (MingLingBio, Cat# P23885). Full‐length *RBM12* and its truncated mutants were constructed by cloning the corresponding sequences into the pcDNA3.1‐Flag vector. The 5’ UTR, CDS, and 3’ UTR of human *SLC7A5* mRNA were inserted into pGL3‐promoter vectors (MingLingBio, Cat# P0194) to generate the luciferase reporter constructs. The *RBM12* promoter region (‐2001 to +100) and the *SLC7A5* promoter region (‐1000 to +100) were inserted into the pGL3‐basic vector (Promega, Cat# E1751) to generate promoter reporter constructs. Point mutations were introduced by site‐directed mutagenesis. RBM12 mutants H34A, K76A, Q80A, I83A, S86A, and E91A were generated using RBM12 as the template and cloned into the pcDNA3.1‐Flag vector. ALKBH5 H204A‐Myc was generated by mutating H204 to A and cloned into the pLVX‐EF1α‐IRES‐Neo vector. Mutant *SLC7A5* 3’ UTR reporter constructs (Mut 1, Mut 2, and Mut 3) were generated by mutating 10 m^6^A sites (A to T) in the Peak 1 region, 18 m^6^A sites (A to T) in the Peak 2 region, and a total of 28 m^6^A sites (A to T) in both Peak 1 and Peak 2 regions of the *SLC7A5* mRNA 3’ UTR, respectively. Mutant RBM12 promoter reporter constructs were generated by mutating TA to GG at either the potential OTX1 binding site 1 (‐1687 to ‐1682), the potential OTX1 binding site 2 (‐902 to ‐897), or both, and then cloned into the pGL3‐basic vector. For lentivirus production, the packaging plasmid psPAX2, envelope plasmid pCL‐VSVG, and lentiviral vector were co‐transfected into 293T cells. The media were collected 72 h post‐transfection, and the lentiviruses were then concentrated by ultracentrifugation.

### 4.6 Immunoblot and Co‐Immunoprecipitation (CoIP) Analyses

For immunoblot analysis, cultured cells were lysed in RIPA buffer supplemented with protease inhibitors for 30 min followed by centrifugation at 15 000×g for 10 min at 4°C. The protein concentration was quantified using the BCA protein assay kit (Biosharp, Cat# BL521A). Equal amounts of protein samples were separated by SDS‐PAGE gels and transferred to PVDF membranes (Millipore Sigma, Cat# IPVH00010). After blocking with 5% skim milk for 1 h at room‐temperature, the membranes were incubated with primary antibody overnight at 4°C followed by HRP‐conjugated secondary antibody for 1 h at room‐temperature. Immunoreactivity was detected by using the BioRad Image Lab system with ECL kit (Biosharp, Cat# BL523B). For CoIP, cells were lysed in IP lysis buffer supplemented with protease inhibitors for 30 min and centrifuged at 15 000×g for 10 min at 4°C. Protein lysates were incubated with primary antibody and protein A/G Plus agarose beads (Santa Cruz, Cat# sc‐2003) overnight at 4°C with gentle rotation. The precipitants were then washed with IP wash buffer three times, boiled in SDS loading buffer, and subjected to immunoblot analysis. A complete list of antibodies, including dilutions, is shown in Table S6.

### 4.7 Immunofluorescent Staining

Cultured cells or tumor sections were fixed in 4% paraformaldehyde for 10 min and permeabilized with 0.3% Triton X‐100 in 1% BSA for 30 min at room‐temperature. The slides were then incubated with primary antibody overnight at 4°C, followed by fluorescently labeled secondary antibodies for 2 h at room‐temperature. Subsequently, the slides were incubated with DAPI for 5 min and then imaged using a Leica DMi8 microscope or an Andor confocal spinning disk mounted on a Nikon inverted microscope Eclipse Ti‐E. The antibodies used, including dilutions, are shown in Table S6.

### 4.8 Cell Viability, Tumorsphere Formation, and In Vitro Limiting Dilution Assays

For the cell viability assay, 1500 cells were seeded into each well of a 96‐well plate. Cell viability was assessed on the indicated days following cell seeding using the Cell Titer‐Glo Luminescent Cell Viability Assay kit (Promega, Cat# G7572) in accordance with the manufacturer's protocol. For the tumorsphere assay, 1500 cells were also seeded into each well of a 96‐well plate. The number and diameter of tumorspheres were analyzed on the sixth day after cell seeding. For in vitro limiting dilution assay, GSCs were plated into 96‐well plates at densities of 0, 10, 20, 30, 40, and 50 cells per well, with 24 replicates for each density. Tumorsphere formation was examined in each well after 6 days and then analyzed using the ELDA software at http://bioinf.wehi.edu.au/software/elda/.

### 4.9 EdU Incorporation Assay

GSCs were incubated with EdU for 1 h and then fixed with 4% paraformaldehyde for 10 min. Subsequently, staining was performed using the BeyoClick EdU Cell Proliferation Kit with Alexa Fluor 555 (Beyotime, Cat# C0075S) according to the manufacturer's instructions.

### 4.10 RNA Stability Assay and Amino Acid Analysis

For the RNA stability assay, GSCs were incubated with 9 µg/mL actinomycin D (GLPBIO, Cat# GL16866) for the indicated timepoints (1, 2, 3, and 4 h). Total RNA was then isolated, and RNA decay was analyzed by qPCR. For amino acid analysis, cells were harvested, resuspended in ddH_2_O, and then subjected to freeze‐thaw cycles using a −80°C freezer. After centrifugation at 15 000 g for 25 min, the supernatant was collected. The cellular amino acid contents were analyzed using an automated amino acid analyzer (membraPure).

### 4.11 Luciferase Reporter and Chromatin Immunoprecipitation (ChIP) Assays

For the luciferase reporter assay, cells were co‐transfected with corresponding plasmids and the pRL internal control vector (Promega, Cat# E2231). To ensure equal amounts of DNA in all transfection combinations, the appropriate control vector was added. The activities of firefly luciferase and Renilla luciferase were measured 48 h post‐transfection using the Dual‐Luciferase Reporter Assay System (Promega, Cat# E1910) according to the manufacturer's instructions. For the ChIP assay, cells were fixed with 1% formaldehyde for 10 min at room‐temperature, and the cross‐linking reaction was terminated with glycine. The chromatin lysates were sonicated to break the chromatin, pre‐cleared with Protein A/G agarose beads (Santa Cruz, Cat# sc‐2003) in the presence of salmon sperm DNA and BSA, and then subjected to immunoprecipitation with the indicated antibody or control IgG overnight. The next day, the lysates were incubated with Protein A/G Plus agarose beads for 1 hour at 4°C with gentle rotation. The beads were then washed and de‐crosslinked in elution buffer at 65°C. DNA was purified using the DNA purification Kit (Omega, Cat# D2500‐02) and analyzed by qPCR. The primers used were as follows: Potential OTX1‐binding site 1, forward 5’‐CACGCCCAGCTAATGTTTGT‐3’, reverse 5’‐TCCACCCTGGATGACAAAAG‐3’; Potential OTX1‐binding site 2, forward 5’‐TTTTGGCTGTTTTGTCAGGA‐3’, reverse 5’‐GTGTCTAGCTTGGCCCTTCA‐3’. The antibodies used are listed in Table S6.

### 4.12 RNA Immunoprecipitation (RIP) and Methylated RNA Immunoprecipitation (MeRIP)

For RIP, cells were fixed with 1% formaldehyde for 10 min at room‐temperature, and the cross‐linking reaction was terminated with glycine. The cell lysates were sonicated to shear the RNA, pre‐cleared with Protein A/G agarose beads (Santa Cruz, Cat# sc‐2003), and then subjected to immunoprecipitation with the indicated antibody and Protein A/G Plus agarose beads overnight at 4°C. The next day, the beads were washed extensively and de‐crosslinked at 65°C. RNA was purified, reverse transcribed, and then analyzed by PCR or qPCR. For MeRIP, total RNA was isolated and then fragmented using fragmentation buffer at 94°C for 4 min. The fragmented RNA was precipitated in ethanol at ‐80°C overnight, allowed to air dry, and resuspended in RNase‐free water. The RNA was immunoprecipitated with an anti‐m^6^A antibody for 6 h at 4°C, followed by incubation with Protein A/G Plus agarose beads for 4 h at 4°C with gentle rotation. The beads were washed thoroughly and incubated in MeRIP elution buffer containing 6 mm m^6^A (MCE, Cat# HY‐111926) for 1 h at 4°C. The RNA was then precipitated with ethanol, reverse transcribed, and then analyzed by qPCR. The primers used for RIP and MeRIP were as follows: *SLC7A5* 3’ UTR Peak 1, forward 5’‐CCCTTCCCTCCTTTGTTTAC‐3’, reverse 5’‐AGGGTTGGTTTTCACGAATG‐3’; *SLC7A5* 3’ UTR Peak 2, forward 5’‐CTCTCAGCAAGTGCCCAGT‐3’, reverse 5’‐GACGCTGTGAAGTCTGTC CA‐3’; β‐actin 3’ UTR: forward 5’‐GTGGCCGAGGACTTTGATTG‐3’, reverse 5’‐CCTGTAACAACGC ATCTCATATT‐3’. The antibodies used for RIP and MeRIP are listed in Table S6.

### 4.13 MeRIP‐seq Data Analysis

MeRIP‐seq data for GSCs were obtained from the GEO database (GSE158741). Raw sequencing reads were processed using Cutadapt v4.3 to remove adapter sequences and low‐quality bases. Subsequently, the cleaned reads were aligned to the human reference genome (GRCh38, NCBI) using STAR (v2.7.10b) with the following parameters: –outFilterMultimapNmax 3, –outFilterMismatchNmax 1, and –outFilterMatchNmin 20. These parameters ensured the selection of uniquely mapped reads with a mapping quality of at least 20 for downstream analysis. To identify m^6^A‐enriched regions, MACS2 v2.2.7.1 was employed, using input samples as controls. The analysis was conducted with default settings, but with the additional parameters ‐q 0.05, ‐nomodel, and ‐call‐summits to disable fragment size estimation and resolve subpeaks within the identified peaks. Finally, a bedgraph file was generated to create the signal track.

### 4.14 Tumor Xenograft Models and In Vivo Treatment

All animal experiments were conducted in compliance with the ARRIVE guidelines and approved by the Animal Experimental Ethics Committee of Huazhong Agricultural University (Approval No. HZAUMO‐2024‐0347). 6‐week‐old female BALB/c nude mice were used and housed in the animal facility of the Laboratory Animal Center at Huazhong Agricultural University. Mice were maintained at 22°C to 24°C under a 12 h light/12 h dark cycle. 50 000 GSCs were implanted into the right cerebral cortex of each mouse at a depth of 3.5 mm to generate intracranial xenografts. Animals were maintained until neurological signs occurred, unless otherwise indicated. For the JPH203 (TargetMol, Cat# T11727) treatment, mice implanted with GSCs for 7 days were then administered with DMSO or 25 mg/kg JPH203 via intraperitoneal injection 6 days per week.

### 4.15 Statistical Analysis

Data are presented as mean ± SD. Statistical analysis was performed using GraphPad Prism 9. For comparisons between two groups, statistical significance was evaluated using a two‐tailed unpaired Student's *t*‐test (for normally distributed data) or Mann–Whitney test (for non‐normally distributed data). For comparisons involving more than two groups, one‐way ANOVA analysis followed by Tukey's test was used to assess statistical significance. For analyses involving two groups with subgroups, two‐way ANOVA analysis followed by Sidak's test was employed. For analyses involving more than two groups with subgroups, two‐way ANOVA analysis followed by Tukey's test was used. Data for all ANOVA analyses were normally distributed. For Kaplan–Meier survival curves, statistical differences were determined using the log‐rank test. *P* < 0.05 was considered statistically significant.

## Author Contributions

H.L., W.L., and S.Z. contributed equally to this work. W.T. and H.L. designed the experiments. H.L., W.L., S.Z., L.W., P.L., Z.H., Z.Q., C.L., M.G., and Z.X. performed the experiments. J.Q., N.Z., J.M., W.Z., Z.D. S.X., Z.Z., and X.W. provided scientific input. W.T. and H.L. analyzed the data and wrote the manuscript.

## Ethics Statement

The studies involving human tissues were conducted in accordance with the principles of the Declaration of Helsinki and approved by the Ethics Committee of Taihe Hospital (Approval No. 2023KS11). Informed consent was obtained from all patients. All animal experiments were conducted in compliance with the ARRIVE guidelines and approved by the Animal Experimental Ethics Committee of Huazhong Agricultural University (Approval No. HZAUMO‐2024‐0347).

## Conflicts of Interest

The authors declare no conflicts of interest.

## Supporting information




**Supporting File 1**: advs76239‐sup‐0001‐SuppMat.docx.

**Supporting File 2**: advs76239‐sup‐0002‐Table.docx.

## Data Availability

The data that support the findings of this study are openly available in National Genomics Data Center, China at http://ngdc.cncb.ac.cn/gsa‐human/browse/HRA012041, reference number HRA012041
