# Supplementary material for: RBM12 Maintains Glioma Stem Cells by Activating Amino Acid‐Dependent mTORC1 Signaling via SLC7A5 mRNA Stabilization
Source: Adv Sci (Weinh). 2026 Jun 22;13(52):e76239. doi: 10.1002/advs.76239 (PMC13336356; doi:10.1002/advs.76239)
Supplement: Supplementary file 1 [file ADVS-13-e76239-s001.docx]

**Table S1. KEGG analysis of downregulated genes after RBM12 knockdown in GSCs compared to control GSCs.**

The top 8 signaling pathways identified by KEGG enrichment analysis of downregulated genes in RBM12-silenced GSCs (H2S).

| KEGGID | Description | GeneRatio | BgRatio | pvalue | GeneName | Count |
| --- | --- | --- | --- | --- | --- | --- |
| hsa04144 | Endocytosis | 6/60 | 232/7161 | 0.012604 | RHOA/WIPF1/SPG21/PLD2/RAB5A/  CHMP3 | 6 |
| hsa04150 | mTOR signaling pathway | 5/60 | 144/7161 | 0.006897 | RHOA/SLC7A5/STRADB/LRP5/FZD2 | 5 |
| hsa04928 | Parathyroid hormone synthesis, secretion and action | 4/60 | 103/7161 | 0.010629 | RHOA/PLD2/LRP5/ADCY6 | 4 |
| hsa04360 | Axon guidance | 4/60 | 173/7161 | 0.056428 | RHOA/NCK2/EFNB3/DPYSL5 | 4 |
| hsa04024 | cAMP signaling pathway | 4/60 | 196/7161 | 0.081207 | RHOA/PLD2/ADORA1/ADCY6 | 4 |
| hsa04962 | Vasopressin-regulated water reabsorption | 3/60 | 43/7161 | 0.00544 | ADCY6/RAB5A/DCTN6 | 3 |
| hsa04071 | Sphingolipid signaling pathway | 3/60 | 43/7161 | 0.00544 | PLD2/RHOA/ADORA1 | 3 |
| hsa05135 | Yersinia infection | 3/60 | 116/7161 | 0.072922 | WIPF1/RHOA/ACTR3B | 3 |

**Table S2. Genes exhibiting differential splicing events after RBM12 knockdown in GSCs.**

Genes with differential alternative splicing events following RBM12 knockdown in GSCs (including alternative 3' start site, alternative 5' start site, mutually exclusive exon, retained intron, and skipped exon) are listed separately below. The alternative splicing events with a false discovery rate (FDR)<0.05 and an absolute value of inclusion level difference (|IncLevelDifference|)>0.1 were considered significant.

**S2.1 Alternative 3' start site:**

| CECR2 | U2AF1L4 | CEP192 | SPG7 | MTHFSD | ZNF19 | CACNA1H | WARS | NEMF |
| --- | --- | --- | --- | --- | --- | --- | --- | --- |
| RBM26-AS1 | TMEM198B | CAPRIN2 | RECQL | MIAT | HPS4 | NFKB2 | WDR11 | BACE1 |
| AKAP8L | RPS6KB2 | SLC25A45 | TICRR | POGLUT1 | RBM39 | TMEM256-PLSCR3 | | GEN1 |
| PABPC1L | CEP76 | GPSM2 | TSACC | ZNF169 | MTMR9LP | BCL6 | LTBP1 | TMEM116 |
| ETNK2 | LETMD1 | PSRC1 | WNK1 | GABBR1 | NUP62 | PKMYT1 | ZNF286A | TRIM39 |
| OGG1 | PHF1 | NPHP1 | DUXAP10 | XRCC4 | SNRPN | GALT | ZNF559 |  |

**S2.2 Alternative 5' start site:**

| ATG4D | PCNT | PPIEL | GCNT2 | ZNF266 | FBRSL1 | CEP164 | BX890604.2 | FXYD2 |
| --- | --- | --- | --- | --- | --- | --- | --- | --- |
| MAP3K3 | SMG7 | PBX4 | UBE2T | TATDN1 | CSAG2 | ANXA2 | UBQLN1 | SCAPER |
| ATXN2L | MSTO1 | UBE2J2 | PRPF38A | C11orf74 | TAZ | CA14 | B4GALT4 | C1orf109 |
| ARHGEF40 | ITGA7 | ATP5PO | CEP170 | BPTF | HSD11B1L | CCDC14 | STK16 | DSCR8 |
| CPNE1 | SH3BP2 | MIB2 | SH3YL1 | PDXK | GIGYF2 | ZSCAN25 | LLGL1 | CTNND1 |
| ULK3 | TTC31 | TRMT11 | MFF | TRERF1 | CHCHD7 | PIGA | ZNF707 | PABPC4 |
| VPS29 | CSAG3 | KALRN |  |  |  |  |  |  |

**S2.3 Mutually exclusive exon:**

| EMID1 | MILR1 | USP54 | HDAC11 | ZNF341 | SUSD1 | ESPL1 | DLEU2 | ZNF783 |
| --- | --- | --- | --- | --- | --- | --- | --- | --- |
| AC012313.3 | NDEL1 | NUMA1 | ARIH2 | DHX35 | CS | NBPF9 | TMEM63C | TADA2A |
| TRPM4 | AL109827.1 | AC068152.1 | AACS | GIGYF2 | ARHGAP4 | CCNB1IP1 | TMBIM6 | RAPH1 |
| AURKA | PKD1P1 | SENP7 | KIF9 | PCBP1-AS1 | WDR27 | CCDC191 | ZDHHC11 | C1QTNF6 |
| PSTPIP2 | ZNF592 | ASPH | SPATA6 | CARF | NSD2 | KANSL3 | LETMD1 | STARD9 |
| PTPN2 | KRBA2 | ST3GAL6 | AKT2 |  |  |  |  |  |

**S2.4 Retained intron:**

| MYO9B | EIF3G | FN1 | WASH6P | AFTPH | PSMB8 | VPS28 | ZMYM2 | NUP62 |
| --- | --- | --- | --- | --- | --- | --- | --- | --- |
| RGL3 | TPT1-AS1 | ABTB1 | CTNNB1 | FAAH | NPHP1 | MINK1 | CACNB3 | EFHC1 |
| SPAG4 | TMEM198B | PCSK4 | PPIEL | SAT2 | DNM1 | TCEAL4 | RABGGTA | ZFAND2B |
| ATP5F1D | CAPRIN2 | DYNC1LI1 | NICN1 | IFRD2 | ZKSCAN1 | NUDT17 | TRPV1 | TMEM218 |
| MAPK7 | B4GALNT3 | OBSL1 | BTBD19 | IRAK1 | JMJD7-PLA2G4B | | HDAC6 | NAGPA |

**S2.5 Skipped exon:**

| PRMT2 | PKD1P1 | AC009533.1 | OBSCN | AGBL5 | PDE9A | MSTO2P | LAS1L |
| --- | --- | --- | --- | --- | --- | --- | --- |
| GATD3A | NTAN1 | MIAT | MAP4 | SCAPER | ZDHHC11 | L3MBTL3 | CAMLG |
| RPL23AP82 | PDXDC1 | TNFRSF19 | CEP57L1 | PHF21A | SMG5 | AC126755.1 | RNF14 |
| APOBEC3D | LINC01569 | HPS4 | HPS1 | STAG3L5P-PVRIG2P-PILRB | | | VPS13B |
| PISD | NAA60 | CTBP2 | FGFR3 | CCDC189 | CCDC125 | SEMA3G | FAM193B |
| DUSP18 | ERVK13-1 | VTI1A | GALE | BX890604.2 | IL17RC | RAB4A | NDUFV3 |
| ZNF256 | MEIOB | NT5C2 | WASHC1 | DNAJC27 | PPP2R5C | CLUHP3 | PHYKPL |
| ZBTB21 | ALKBH6 | ZFYVE27 | ZNF429 | PPP1R12B | FAM86B2 | NFKBIZ | MFSD14C |
| ZNF761 | CHTF18 | EXOC7 | EYA3 | GPSM2 | NME6 | WDR17 | AC138035.1 |
| FUZ | METTL26 | NUTM2B-AS1 | ZBTB38 | PALB2 | FER | COQ8A | YWHAH |
| TRPM4 | CHSY1 | CATSPER2 | NSL1 | UBQLN1 | RWDD3 | MFF | AKAP9 |
| GEMIN7 | ZNF592 | ASAH2B | IFT122 | MPPE1 | GLRB | LETMD1 | ST6GALNAC6 |
| DPY19L3 | UBE2Q2P2 | MARCHF8 | KPNA1 | AL358113.1 | RCC1 | LIMK2 | LRRC23 |
| IZUMO4 | ZFAND6 | CCNYL2 | TNFAIP2 | RAPGEF2 | SCLT1 | DTNB | PSMD6 |
| SLC25A41 | AC087632.1 | HINFP | AP1G1 | CROCCP2 | HEXIM2 | ANKRD11 | HCG18 |
| MTMR3 | RNF111 | PHLDB1 | PCSK4 | METTL5 | SLMAP | CEP152 | MDC1 |
| PKIG | DNAAF4 | PCSK7 | N4BP2 | C9orf85 | RTKN | GPR157 | AC006453.2 |
| CCNE1 | PPIP5K1 | CACNB3 | GLT8D1 | SLC38A9 | ZNF815P | AC093827.4 | NASP |
| KIAA1755 | PDCD6IPP2 | PMM1 | PHF7 | NADSYN1 | PHACTR4 | TROVE2 | DENND2A |
| DZANK1 | SPOP | PPP6R3 | RUVBL1 | TPM4 | MATN1-AS1 | NKTR | POLR2J4 |
| CSNK2A1 | SLC24A1 | RPS6KB2 | RBM6 | THEMIS2 | ROBO1 | PACRGL | KCTD20 |
| CCT8 | KIAA0586 | KDM2A | ARHGAP11A | CMTR2 | MRPL55 | KCTD9 | LPIN1 |
| RPRD1A | TMEM260 | FRMD8 | CCDC66 | MSH5 | GUSBP11 | TMEM241 | SEPT4 |
| TRAPPC8 | MAPK1IP1L | SLC25A45 | BCHE | YAP1 | CYP20A1 | BCL11A | ANKRD6 |
| FAM210A | AL365295.1 | ESRRA | CAPN7 | HSD11B1L | CAP1 | CROCC | TCTN1 |
| ABCA5 | DCAF11 | VWCE | ITGA7 | CEP44 | ZNF691 | C11orf65 | MED12L |
| LARGE1 | ARHGEF40 | ABCC4 | TTLL3 | ELN | ZGRF1 | TRPS1 | ERBB2 |
| FLRT3 | NDRG2 | SLC43A1 | CCDC191 | EIF4G1 | TTC3 | AC105052.3 | ABL2 |
| CORO6 | ANKRD10 | NT5DC3 | TAF1 | MIR4435-2HG | SCMH1 | WWP1 | CS |
| FLOT2 | DLEU2 | PIK3IP1 | SMN1 | RHOT1 | TBCE | LIPT1 | CAMK2G |
| SPECC1 | SPRYD7 | STK33 | MEF2A | ATP5S | AL392172.1 | SCLY | TIAM2 |
| EPN2 | CPNE1 | PGAP2 | MICAL1 | LINC01535 | USP47 | NEDD4L | WDR27 |
| SENP3-EIF4A1 | | PEAK1 | RHOC | ATG12 | AL512353.1 | TNK2 | ADAP1 |
| RNF167 | AC156455.1 | ZNF516 | FBF1 | MFSD8 | CLIP1 | MTMR12 | PDGFC |
| SPG7 | RNF34 | DMPK | AMZ2P1 | TMEM253 | GIGYF2 | ZNF266 | PSMG3-AS1 |
| BCAR1 | MAPKAPK5 | SIRT6 | MGAT5 | CROCCP3 | POLQ | KMT2C | MAD1L1 |
| AC010547.4 | VPS29 | ANAPC10 | FIGNL1 | PYM1 | BRWD3 | GTF2H2 | TBL1XR1 |
| WWP2 | LTA4H | LMLN | PABPC1L | RABGGTB | RMDN1 | FAM86EP | UMAD1 |
| NFAT5 | VEZT | FAHD2CP | ATL2 | RAPGEF6 | GAS5 | ACTN2 | PSPH |
| AL109827.1 | PSMA3-AS1 | FNTB | POTEE | LINC00337 | MBOAT2 | MFSD3 | ASPM |
| ZNF720 | RACGAP1 | TUSC3 | IMMP1L | KLHL24 | SPP1 | SCNN1D | HOTAIRM1 |
| LAT | SPATS2 | AFMID | FAR2P2 | SYNGAP1 | NEDD1 | POLB | AC108010.1 |
| ATXN2L | VDR | TMX3 | TBRG1 | AKT2 | TSEN15 | THAP8 | TBC1D31 |
| XPO6 | TM7SF3 | ZNF823 | XPO4 | MST1P2 | TMEM116 | YTHDC2 | POLR2J3 |
| ZNF324B | GOLT1B | LCORL | PCNX2 | NAV2 | BROX | CCDC112 | MECP2 |
| EP400 | SGCE | LINC00963 | PABPC4 | TRPC1 | ZNF618 | RGS3 | KLHDC10 |
| NUDT13 | CENPK | AC004890.2 | SPIN3 | USPL1 | TUT7 | BRAF | BRSK2 |
| NUDCD3 | PILRB | KRBA1 | IP6K2 | STAU2 | SPTLC1 | UROS | GXYLT1 |
| ATXN2 | ATXN7L1 | REPIN1 | DZIP1L | TP53INP1 | FXYD6 | BTN2A3P | SNHG21 |
| LINC00174 | NRF1 | ATG9B | ICE2 | ZNF880 | NRBP2 | GBA2 | CKLF |
| POM121 | CEP41 | LMBR1 | STAG2 | LYRM1 | SLC2A8 | ZSCAN25 | PPARGC1B |
| C1QTNF6 | TTC26 | PIGA | LTBP4 | PVT1 | AFDN | DHRSX |  |

**Table S3. Predicted m6A sites in the *SLC7A5* mRNA 3’ UTR identified by the SRAMP tool.**

| # | Position | Sequence context | Score (binary) | Score (knn) | Score (spectrum) | Score (combined) | Decision | In Peak 1 or Peak 2 |
| --- | --- | --- | --- | --- | --- | --- | --- | --- |
| 1 | 1768 | GGCAG UGGAG GCUGC  UGUGA AA**A**CU CUGGU  ACGAA UCUCA UCCCU | 0.801 | 0.536 | 0.771 | 0.775 | m^6^A site (Very high confidence) | N/A |
| 2 | 2013 | CAAGG GCCCA GACCC  UGGGC AA**A**CA GAGCU  ACUGA GACUU GGAAC | 0.767 | 0.214 | 0.909 | 0.796 | m^6^A site (Very high confidence) | In Peak 1 |
| 3 | 2027 | CUGGG CAAAC AGAGC  UACUG AG**A**CU UGGAA  CCUCA UUGCU ACCAC | 0.856 | 0.611 | 0.898 | 0.86 | m^6^A site (Very high confidence) | In Peak 1 |
| 4 | 2034 | AACAG AGCUA CUGAG  ACUUG GA**A**CC UCAUU  GCUAC CACAG ACUUG | 0.741 | 0.43 | 0.901 | 0.789 | m^6^A site (Very high confidence) | In Peak 1 |
| 5 | 2052 | UGGAA CCUCA UUGCU  ACCAC AG**A**CU UGCAC  UGAAG CCGGA CAGCU | 0.886 | 0.64 | 0.886 | 0.874 | m^6^A site (Very high confidence) | In Peak 1 |
| 6 | 2069 | CACAG ACUUG CACUG  AAGCC GG**A**CA GCUGC  CCAGA CACAU GGGCU | 0.842 | 0.599 | 0.89 | 0.849 | m^6^A site (Very high confidence) | In Peak 1 |
| 7 | 2081 | CUGAA GCCGG ACAGC  UGCCC AG**A**CA CAUGG  GCUUG UGACA UUCGU | 0.768 | 0.313 | 0.885 | 0.792 | m^6^A site (Very high confidence) | In Peak 1 |
| 8 | 2096 | UGCCC AGACA CAUGG  GCUUG UG**A**CA UUCGU  GAAAA CCAAC CCUGU | 0.791 | 0.377 | 0.876 | 0.804 | m^6^A site (Very high confidence) | In Peak 1 |
| 9 | 2108 | UGGGC UUGUG ACAUU  CGUGA AA**A**CC AACCC  UGUGG GCUUA UGUCU | 0.73 | 0.078 | 0.878 | 0.756 | m^6^A site (Very high confidence) | In Peak 1 |
| 10 | 2243 | GAGGU GCUUG AGGCC  CCGAU GG**A**CU CCUGA  CCAUA AUCCU AGCCC | 0.882 | 0.726 | 0.783 | 0.835 | m^6^A site (Very high confidence) | In Peak 1 |
| 11 | 2290 | AGACA CCAUC CUGAG  CCAGG GA**A**CA GCCCC  AGGGU UGGGG GGUGC | 0.766 | 0.267 | 0.803 | 0.756 | m^6^A site (Very high confidence) | In Peak 1 |
| 12 | 3207 | ACCAU CCAGU GGGCC  CGGAG AA**A**CC UGAUG  AACAG UUUGG GGACU | 0.745 | 0.379 | 0.841 | 0.765 | m^6^A site (Very high confidence) | In Peak 2 |
| 13 | 3216 | UGGGC CCGGA GAAAC  CUGAU GA**A**CA GUUUG  GGGAC UCAGG ACCAG | 0.836 | 0.678 | 0.86 | 0.837 | m^6^A site (Very high confidence) | In Peak 2 |
| 14 | 3227 | AAACC UGAUG AACAG  UUUGG GG**A**CU CAGGA  CCAGA UGUCC GUCUC | 0.885 | 0.664 | 0.866 | 0.866 | m^6^A site (Very high confidence) | In Peak 2 |
| 15 | 3234 | AUGAA CAGUU UGGGG  ACUCA GG**A**CC AGAUG  UCCGU CUCUC UUGCU | 0.79 | 0.55 | 0.877 | 0.813 | m^6^A site (Very high confidence) | In Peak 2 |
| 16 | 3311 | CGUUG CUUCC CGCUG  CACAU GG**A**CA GACUU  CACAG CGUCU GCUCA | 0.868 | 0.536 | 0.866 | 0.851 | m^6^A site (Very high confidence) | In Peak 2 |
| 17 | 3315 | GCUUC CCGCU GCACA  UGGAC AG**A**CU UCACA  GCGUC UGCUC AUAGG | 0.875 | 0.483 | 0.871 | 0.854 | m^6^A site (Very high confidence) | In Peak 2 |
| 18 | 3379 | ACGAA UUCCA CUCGU  CCAAG GG**A**CA GCCCA  CGGUC UGGAG GCCGA | 0.852 | 0.646 | 0.852 | 0.841 | m^6^A site (Very high confidence) | In Peak 2 |
| 19 | 3404 | GCCCA CGGUC UGGAG  GCCGA GG**A**CC ACCAG  CAGGC AGGUG GACUG | 0.796 | 0.279 | 0.876 | 0.802 | m^6^A site (Very high confidence) | In Peak 2 |
| 20 | 3423 | AGGAC CACCA GCAGG  CAGGU GG**A**CU GACUG  UGUUG GGCAA GACCU | 0.908 | 0.672 | 0.853 | 0.874 | m^6^A site (Very high confidence) | In Peak 2 |
| 21 | 3427 | CCACC AGCAG GCAGG  UGGAC UG**A**CU GUGUU  GGGCA AGACC UCUUC | 0.875 | 0.564 | 0.898 | 0.869 | m^6^A site (Very high confidence) | In Peak 2 |
| 22 | 3442 | UGGAC UGACU GUGUU  GGGCA AG**A**CC UCUUC  CCUCU GGGCC UGUUC | 0.759 | 0.218 | 0.831 | 0.761 | m^6^A site (Very high confidence) | In Peak 2 |
| 23 | 3483 | GUUCU CUUGG CUGCA  AAUAA GG**A**CA GCAGC  UGGUG CCCCA CCUGC | 0.857 | 0.285 | 0.783 | 0.799 | m^6^A site (Very high confidence) | In Peak 2 |
| 24 | 3541 | GUGUG AAUCC AGGAG  GCAGU GG**A**CA UCGUA  GGCAG CCACG GCCCC | 0.843 | 0.609 | 0.793 | 0.811 | m^6^A site (Very high confidence) | In Peak 2 |
| 25 | 3715 | AACCU CCUGC UCUGG  GACGU GG**A**CA UGCCU  CAAGG AUACA GGGAG | 0.843 | 0.71 | 0.786 | 0.813 | m^6^A site (Very high confidence) | In Peak 2 |
| 26 | 4022 | GGGGC CGUGU CCCGC  GGUGC UG**A**CU GAGGC  CUGCU UCCCC CUCCC | 0.81 | 0.404 | 0.816 | 0.792 | m^6^A site (Very high confidence) | In Peak 2 |
| 27 | 4073 | GCUGU GCUGG AAUUC  CACAG GG**A**CC AGGGC  CACCG CAGGG GACUG | 0.829 | 0.562 | 0.831 | 0.816 | m^6^A site (Very high confidence) | In Peak 2 |
| 28 | 4092 | GGGAC CAGGG CCACC  GCAGG GG**A**CU GUCUC  AGAAG ACUUG AUUUU | 0.908 | 0.612 | 0.855 | 0.872 | m^6^A site (Very high confidence) | In Peak 2 |
| 29 | 4105 | CCGCA GGGGA CUGUC  UCAGA AG**A**CU UGAUU  UUUCC GUCCC UUUUU | 0.822 | 0.522 | 0.846 | 0.816 | m^6^A site (Very high confidence) | In Peak 2 |

**Table S****4. Predicted transcription factors binding to the *RBM12* promoter.**

The *RBM12* promoter region (−2000 bp to +100 bp) was analyzed via the JASPAR database to predict potential transcription factors and their corresponding binding motifs. The top twenty transcription factors, ranked according to their scores, are as follows.

| Protein Name | Score | Sequence |
| --- | --- | --- |
| ZNF460 | 26.82612 | GCCTCAGCCTCCCGAG |
| KLF2 | 14.87796 | CACCACGCCCA |
| KLF6 | 14.58953 | CCACGCCCA |
| ZNF417 | 13.5583 | GGCGCCA |
| LMX1A | 13.18621 | TTAATTA |
| LBX1 | 12.97246 | TTAATTAG |
| ZBTB12 | 12.49126 | CTGGAAC |
| OTX2 | 12.45997 | GGGATTA |
| SP5 | 11.88234 | CCTCCC |
| RBPJ | 11.67997 | TGGGAA |
| PITX1 | 11.66542 | TAATCC |
| SMAD2 | 11.57881 | CCAGAC |
| CREB1 | 11.56856 | TGACGTCA |
| NFATC2 | 11.35969 | TTTTCCA |
| HOXA2 | 11.30474 | TAATTA |
| EMX1 | 11.23351 | TAATTA |
| EMX2 | 11.20782 | TAATTA |
| POU6F1 | 11.1802 | TAATTA |
| GSC | 10.99365 | TAATCC |
| OTX1 | 10.93749 | TAATCC |

**Table S5. shRNA sequences, PCR primers, and qPCR primers used in this study.**

| shRNA / Gene | Sequence |
| --- | --- |
| shNT | Forward: 5’-CCGGCAACAAGATGAAGAGCACCAATTCAAGAGATTGGTGCTCTTCAT  CTTGTTGTTTTTTGGTACC-3’ |
|  | Reverse: 5’-AATTGGTACCAAAAAACAACAAGATGAAGAGCACCAATCTCTTGAATTGG  TGCTCTTCATCTTGTTG-3’ |
| shRBM12-1 | Forward: 5’-CCGGCCATTTAACTTTCCTGGTAATTTCAAGAGAATTACCAGGAAAGTTA  AATGGTTTTTTGGTACC-3’ |
|  | Reverse: 5’-AATTGGTACCAAAAAACCATTTAACTTTCCTGGTAATTCTCTTGAAATTAC  CAGGAAAGTTAAATGG-3’ |
| shRBM12-2 | Forward: 5’-CCGGTACTTGAAAGGGCTACCATTTTTCAAGAGAAAATGGTAGCCCTTT  CAAGTATTTTTTGGTACC-3’ |
|  | Reverse: 5’-AATTGGTACCAAAAAATACTTGAAAGGGCTACCATTTTCTCTTGAAAAAT GGTAGCCCTTTCAAGTA-3’ |
| shRBM12-3’ | Forward: 5’-CCGGTACGTGTTAGTCCTGTTATTTTTCAAGAGAAAATAACAGGACTAAC ACGTATTTTTTGGTACC-3’ |
|  | Reverse: 5’-AATTGGTACCAAAAAATACGTGTTAGTCCTGTTATTTTCTCTTGAAAAATA ACAGGACTAACACGTA-3’ |
| shSLC7A5-1 | Forward: 5’-CCGGGCATTATACAGCGGCCTCTTTTTCAAGAGAAAAGAGGCCGCTGTA TAATGCTTTTTTGGTACC-3’ |
|  | Reverse: 5’-AATTGGTACCAAAAAAGCATTATACAGCGGCCTCTTTTCTCTTGAAAAAG AGGCCGCTGTATAATGC-3’ |
| shSLC7A5-2 | Forward: 5’-CCGGGCCGTGGACTTCGGGAACTATTTCAAGAGAATAGTTCCCGAAGTC CACGGCTTTTTTGGTACC-3’ |
|  | Reverse: 5’-AATTGGTACCAAAAAAGCCGTGGACTTCGGGAACTATTCTCTTGAAATAG TTCCCGAAGTCCACGGC-3’ |
| shALKBH5-1 | Forward: 5’-CCGGGATGAAATCACTCACTGCATATTCAAGAGATATGCAGTGAGTGATT TCATCTTTTTTGGTACC-3’ |
|  | Reverse: 5’-AATTGGTACCAAAAAAGATGAAATCACTCACTGCATATCTCTTGAATATGC AGTGAGTGATTTCATC-3’ |
| shALKBH5-2 | Forward: 5’-CCGGCCACCCAGCTATGCTTCAGATTTCAAGAGAATCTGAAGCATAGCT GGGTGGTTTTTTGGTACC-3’ |
|  | Reverse 5’-AATTGGTACCAAAAAACCACCCAGCTATGCTTCAGATTCTCTTGAAATCT GAAGCATAGCTGGGTGG-3’ |
| shALKBH5-3’ | Forward: 5’-CCGGAGGTTCTCATATTCTTGGTATTTCAAGAGAATACCAAGAATATGAG AACCTTTTTTTGGTACC-3’ |
|  | Reverse: 5’-AATTGGTACCAAAAAAAGGTTCTCATATTCTTGGTATTCTCTTGAAATACC AAGAATATGAGAACCT-3’ |
| shYTHDF2 | Forward: 5’-CCGGCCACAGGCAAGGCCCAATAATTTCAAGAGAATTATTGGGCCTTGC CTGTGGTTTTTTGGTACC-3’ |
|  | Reverse: 5’-AATTGGTACCAAAAAACCACAGGCAAGGCCCAATAATTCTCTTGAAATTA TTGGGCCTTGCCTGTGG-3’ |
| shOTX1-1 | Forward: 5’-CCGGTGTCTGGACTATAAGGACCAATTCAAGAGATTGGTCCTTATAGTCC AGACATTTTTTGGTACC-3’ |
|  | Reverse: 5’-AATTGGTACCAAAAAATGTCTGGACTATAAGGACCAATCTCTTGAATTGG TCCTTATAGTCCAGACA-3’ |
| shOTX1-2 | Forward: 5’-CCGGGCCTAGCAACACCTCGTGTATTTCAAGAGAATACACGAGGTGTTG CTAGGCTTTTTTGGTACC-3’ |
|  | Reverse: 5’-AATTGGTACCAAAAAAGCCTAGCAACACCTCGTGTATTCTCTTGAAATAC ACGAGGTGTTGCTAGGC-3’ |
| *18S rRNA*  (PCR, qPCR) | Forward 5’-CAGCCACCCGAGATTGAGCA-3’ |
|  | Reverse 5’-TAGTAGCGACGGGCGGTGTG-3’ |
| *SLC7A5*  (PCR, qPCR) | Forward 5’-CCGTGAACTGCTACAGCGT-3’ |
|  | Reverse 5’-CTTCCCGATCTGGACGAAGC-3’ |
| *RHOA*  (PCR, qPCR) | Forward 5’-GATTGGCGCTTTTGGGTACAT-3’ |
|  | Reverse 5’-AGCAGCTCTCGTAGCCATTTC-3’ |
| *STRADB*  (PCR, qPCR) | Forward 5’-TGGCCTATGGTTCAGCAAGTC-3’ |
|  | Reverse 5’-AAATCATACACAGCCCTATGCC-3’ |
| *LRP5*  (PCR, qPCR) | Forward 5’-ACTCGCTGTGAGGAGGACAAT-3’ |
|  | Reverse 5’-GGCAGGCGCATGTGTAGAA-3’ |
| *FZD2*  (PCR) | Forward 5’-GTGCCATCCTATCTCAGCTACA-3’ |
|  | Reverse 5’-CTGTTCATCGTGTGGTACGTG-3’ |
| *RBM12*  (qPCR) | Forward 5’-GGGTGAGGCTTTCATCGTTTT-3’ |
|  | Reverse 5’-CGCCTACGACTCAGTTCAATC-3’ |
| *SLC3A2*  (qPCR) | Forward 5’-TGAATGAGTTAGAGCCCGAGA-3’ |
|  | Reverse 5’-GTCTTCCGCCACCTTGATCTT-3’ |
| *ALKBH5*  (qPCR) | Forward 5’-AGTTCCAGTTCAAGCCTATTCG-3’ |
|  | Reverse 5’-TGAGCACAGTCACGCTTCC-3’ |
| *YTHDF2*  (qPCR) | Forward 5’-AGCCCCACTTCCTACCAGATG-3’ |
|  | Reverse 5’-TGAGAACTGTTATTTCCCCATGC-3’ |
| *NEAT1*  (qPCR) | Forward 5’-CCAGTTTTCCGAGAACCAAA-3’ |
|  | Reverse 5’-ATGCTGATCTGCTGCGTATG-3’ |
| *GAPDH*  (qPCR) | Forward 5’-TGCCTCCTGCACCACCAACT-3’ |
|  | Reverse 5’-CGCCTGCTTCACCACCTTC-3’ |
| *ZBTB12*  (qPCR) | Forward 5’-AACGCTACGGAACATGAACCA-3’ |
|  | Reverse 5’-CCTTGTGGCCTCGAAACTTGA-3’ |
| *NFATC2*  (qPCR) | Forward 5’-GAGCCGAATGCACATAAGGTC-3’ |
|  | Reverse 5’-CCAGAGAGACTAGCAAGGGG-3’ |
| *OTX1*  (qPCR) | Forward 5’-GCGTCGTCGCTGAGTACAC-3’ |
|  | Reverse 5’-ACATGGGATAAGAGGCTGCTG-3’ |
|  | |

**Table S6.** **List of primary antibodies used in this study.**

| Antibodies | Source | Catalog | Application | Dilution |
| --- | --- | --- | --- | --- |
| RBM12 | Novus Biologicals | NBP2-13208 | IF | 1:100 |
| SOX2 | R&D | AF2018 | IF | 1:100 |
| SOX2 | Santa Cruz | sc-365964 | IF | 1:100 |
| Ki67 | Cell Signaling | 9129S | IF | 1:400 |
| SLC7A5 | Proteintech | 28670-1-AP | IF | 1:250 |
| OTX1 | Boster Biologicals | BM4103 | IF | 1:100 |
| Flag | Proteintech | 20543-1-AP | IF | 1:300 |
| RBM12 | Santa Cruz | sc-514258 | WB | 1:400 |
| SOX2 | Santa Cruz | sc-365964 | WB | 1:1000 |
| Myc | Proteintech | 60003-2-Ig | WB | 1:5000 |
| Myc | ABclonal | AE009 | WB | 1:3000 |
| Flag | ABclonal | AE005 | WB | 1:2000 |
| Flag | ABclonal | AE092 | WB | 1:2500 |
| Tubulin | ABclonal | AC012 | WB | 1:10000 |
| GFAP | ABclonal | A19058 | WB | 1:1000 |
| pS6 | Proteintech | 29223-1-AP | WB | 1:1000 |
| S6 | Proteintech | 14823-1-AP | WB | 1:1000 |
| p4E-BP1 | ABclonal | AP0030 | WB | 1:1000 |
| 4E-BP1 | ABclonal | A1248 | WB | 1:1500 |
| Puromycin | ABclonal | A21205 | WB | 1:5000 |
| SLC7A5 | Proteintech | 28670-1-AP | WB | 1:1500 |
| ALKBH5 | ABclonal | A22137 | WB | 1:1000 |
| FTO | ABclonal | A20992 | WB | 1:1000 |
| OTX1 | Boster Biologicals | BM4103 | WB | 1:1000 |
| GAPDH | ABclonal | AC033 | WB | 1:15000 |
| Cleaved Caspase-7 | Affinity Biologicals | AF4023 | WB | 1:1000 |
| Cleaved PARP | ABclonal | A22535 | WB | 1:1000 |
| SLC3A2 | ABclonal | A24735 | WB | 1:1000 |
| YTHDF2 | ABclonal | A24287 | WB | 1:1000 |
| Flag | ABclonal | AE005 | IP | N/A |
| Flag | ABclonal | AE092 | IP | N/A |
| Myc | Proteintech | 60003-2-Ig | IP | N/A |
| Myc | ABclonal | AE009 | IP | N/A |
| Flag | ABclonal | AE092 | ChIP | N/A |
| m^6^A | ABclonal | A22411 | MeRIP | N/A |
| RBM12 | Santa Cruz | sc-514259 | RIP  N/A | N/A |
| ALKBH5 | Proteintech | 16837-1-AP | RIP | N/A |
| YTHDF2 | Proteintech | 24744-1-AP | RIP | N/A |
