# Supplementary material for: RBM12 Maintains Glioma Stem Cells by Activating Amino Acid‐Dependent mTORC1 Signaling via SLC7A5 mRNA Stabilization
Source: Adv Sci (Weinh). 2026 Jun 22;13(52):e76239. doi: 10.1002/advs.76239 (PMC13336356; doi:10.1002/advs.76239)
Supplement: Supplementary file 2 [file ADVS-13-e76239-s002.docx]

Supplemental Figures for

**RBM12 Maintains** **Glioma Stem Cells by Activating Amino Acid-Dependent mTORC1 Signaling via *SLC7A5* mRNA Stabilization**

Hong Lei *et al.*

**Figure S1. RBM12 promotes GSC proliferation and self-renewal.**

(A-E) Analysis of *RBM12* mRNA expression in human NTB tissues and GBM samples from the TCGA HG-U133A (A), TCGA RNA-seq (B), Rembrandt (C), Murat (D), and Kamoun (E) databases. TCGA HG-U133A database: NTBs, n=10; GBMs, n=528. TCGA RNA-seq database: NTBs, n=4; GBMs, n=156. Rembrandt database: NTBs, n=28; GBMs, n=219. Murat database: NTBs, n=4; GBMs, n=80. Kamoun database: NTBs, n=9; GBMs, n=11.

(F) Analysis of RBM12 protein expression in human NTB tissues and GBM samples from the CPTAC database. NTBs, n=10; GBMs, n=100.

(G) Immunoblot analysis of RBM12 expression in NPCs and GSCs.

(H) EdU incorporation assay of GSCs transduced with shNT or shRBM12. Scale bar, 60 μm.

(I) Quantification of the fraction of EdU^+^ cells in GSCs expressing shNT or shRBM12. n=3.

(J) Immunoblot analysis of RBM12 expression in NSTCs transduced with shNT or shRBM12.

(K) Cell viability assay of NSTCs transduced with shNT or shRBM12. n=4.

(L) Immunoblot analysis of RBM12 expression in GSCs transduced with vector control or RBM12-Flag.

(M) Cell viability assay of GSCs transduced with vector control or RBM12-Flag. n=4.

(N) Representative images of tumorspheres derived from GSCs expressing vector control or RBM12-Flag. Scale bar, 80 μm.

(O and P) Quantification of the diameter (O) and number (P) of tumorspheres derived from GSCs expressing vector control or RBM12-Flag. n=4.

(Q) In vitro limiting dilution assay of the tumorsphere formations of GSCs expressing vector control or RBM12-Flag.

Data information: Data are presented as mean ± SD. **P*<0.05, ***P*<0.01, ****P*<0.001, *****P*<0.0001, two-tailed unpaired *t*-test (B, C, E, O, and P), Mann-Whitney test (A, D, and F), one-way ANOVA analysis followed by Tukey’s test (I), two-way ANOVA analysis followed by Tukey’s test (K), two-way ANOVA analysis followed by Sidak's test (M), and ELDA analysis for differences in stem cell frequencies (Q).

**Figure S2.** **RBM12 promotes the growth of GSCs across three subtypes**

(A) Immunoblot analysis of RBM12 expression in six patient-derived GSCs across three subtypes.

(B) Immunoblot analysis of RBM12 expression in GSCs (T4121 and T3264) transduced with shNT or shRBM12.

(C) Immunoblot analysis of RBM12 expression in GSCs (T3691, T3832, and T3028) transduced with shNT or shRBM12.

(D) Cell viability assay of five patient-derived GSCs from three subtypes transduced with shNT or shRBM12. n=4.

Data information: Data are shown as mean ± SD. *****P*<0.0001, two-way ANOVA analysis followed by Tukey’s test (D).

**Figure S3. SLC7A5 is enriched in GSCs.**

(A) PCR analysis of *RHOA*, *SLC7A5*, *STRADB*, *LRP5* and *FZD2* expression in GSCs.

(B-E) qPCR analysis of *SLC7A5* (B), *RHOA* (C), *STRADB* (D), and *LRP5* (E) expression in five pairs of matched GSCs and NSTCs. n=3.

(F) qPCR analysis of *SLC7A5* expression in GSCs transduced with vector control or RBM12-Flag. n=3.

(G) qPCR analysis of *SLC3A2* expression in GSCs transduced with shNT or shRBM12. n=3.

(H) qPCR analysis of *SLC3A2* expression in GSCs transduced with vector control or RBM12-Flag. n=3.

(I) Immunoblot analysis of SLC7A5 and ALKBH5 expression during serum-induced GSC differentiation.

(J) qPCR analysis of *RBM12* and *ALKBH5* mRNA levels in GSCs transduced with shNT or shSLC7A5. n=3.

(K) Immunoblot analysis of RBM12 and ALKBH5 protein levels in GSCs transduced with shNT or shSLC7A5.

Data information: Data are presented as mean ± SD. ****P*<0.001, *****P*<0.0001, two-tailed unpaired *t*-test (B-F and H), one-way ANOVA analysis followed by Tukey’s test (G and J).

**Figure S4. RBM12 promotes GSC maintenance through SLC7A5-mTORC1 pathway.**

(A) CoIP assays of interaction between SLC7A5-Flag and SLC3A2 in GSCs expressing SLC7A5-Flag. The precipitated proteins and total lysates were then analyzed by immunoblotting using the indicated antibodies.

(B and C) Intracellular levels of non-SLC7A5-substrate amino acids in GSCs (H2S, A, and T387, B) expressing shNT, shRBM12-1, shRBM12-1+Vector, or shRBM12-1+SLC7A5. n=3.

(D) Representative images of tumorspheres derived from GSCs expressing shNT, shRBM12-1, shRBM12-1+Vector, or shRBM12-1+SLC7A5. Scale bar, 80 μm.

(E) Immunofluorescent staining of SOX2 (red) in tumor xenografts derived from GSCs expressing shNT, shRBM12-1, shRBM12-1+Vector, or shRBM12-1+SLC7A5. GBM xenografts were collected from mice when neurological signs occur after GSC transplantation. Scale bar, 50 μm.

(F) Quantification of SOX2^+^ cells in tumor xenografts derived from GSCs expressing shNT, shRBM12-1, shRBM12-1+Vector, or shRBM12-1+SLC7A5. n=5 tumors per group.

(G) Immunoblot analysis of pS6, S6, p4E-BP1, 4E-BP1, and RBM12 protein levels in GSCs treated with 5 nM Rapamycin or DMSO in combination with RBM12-Flag or vector control for two days.

(H) Cell viability assay of GSCs treated with 5 nM Rapamycin or DMSO in combination with RBM12-Flag or vector control for six days. n=4.

(I) In vitro limiting dilution assay of the tumorsphere formations of GSCs treated with 5 nM Rapamycin or DMSO in combination with RBM12-Flag or vector control for six days.

Data information: Data are shown as mean ± SD. **P*<0.05, ***P*<0.01, ****P*<0.001, *****P*<0.0001, one-way ANOVA analysis followed by Tukey’s test (B, C, F, and H), and ELDA analysis for differences in stem cell frequencies (I).

**Figure S5. RBM12 stabilizes *SLC7A5* transcripts via m^6^A demethylation.**

(A) Reporter gene assays were performed in HEK293T cells transfected with the *SLC7A5* promoter luciferase reporter (-1000 to +100) and either RBM12-Flag or vector control. n=3.

(B) RNA-seq reads for *SLC7A5* from two shNT and shRBM12 samples.

(C) GSCs expressing vector control or RBM12-Flag were treated with 9 μg/ml ActD and then harvested at the indicated times for qPCR analysis. n=3.

(D) qPCR analysis of the distribution of *SLC7A5, GAPDH* mRNA, and *NEAT1* RNA in subcellular fractions from GSCs expressing shNT or shRBM12. *GAPDH* mRNA is predominantly located in the cytoplasm and *NEAT1* RNA mainly in the nucleus, serving as markers for cytoplasmic and nuclear RNA, respectively. n=3.

(E) qPCR analysis of the distribution of *SLC7A5, GAPDH* mRNA, and *NEAT1* RNA in subcellular fractions from GSCs expressing vector control or RBM12-Flag. n=3.

(F) Top: MeRIP-seq analysis from GSE158741 dataset shows the relative abundance of m^6^A sites along the *SLC7A5* mRNA in GSCs (T387). Bottom: Identification of m^6^A peaks in the 3’ UTR of *SLC7A5*.

(G) RIP-qPCR analysis of RBM12 enrichment at two m^6^A peak regions in the *SLC7A5* 3’ UTR of GSCs expressing vector control or RBM12-Flag. n=3.

(H) MeRIP-qPCR analysis of m^6^A enrichment at two m^6^A peak regions in the *SLC7A5* 3’ UTR of GSCs expressing vector control or RBM12-Flag. n=3.

Data information: Data are presented as mean ± SD. **P*<0.05, ****P*<0.001, *****P*<0.0001, two-tailed unpaired *t*-test (A, G, and H), two-way ANOVA analysis followed by Sidak's test (C), and one-way ANOVA analysis followed by Tukey’s test (D and E).

**Figure S6. RBM12 interacts with ALKBH5 to stabilize the *SLC7A5* transcripts.**

(A) Reciprocal CoIP assays were performed to examine the interaction between exogenous RBM12-Flag and ALKBH5-Myc in HEK293T cells. The precipitated proteins and total lysates were then analyzed by immunoblotting using the indicated antibodies.

(B) qPCR analysis of *SLC7A5* and *ALKBH5* expression in GSCs transduced with shNT or shALKBH5. n=3.

(C) Immunoblot analysis of SLC7A5 and ALKBH5 expression in GSCs transduced with shNT or ALKBH5.

(D) GSCs expressing shNT or shALKBH5 were treated with 9 μg/ml ActD and then harvested at the indicated times for qPCR analysis. n=3.

(E) RIP-qPCR analysis of ALKBH5 enrichment at two m^6^A peak regions in the *SLC7A5* 3’ UTR of GSCs expressing shNT or shALKBH5. n=3.

(F) RIP-qPCR analysis of ALKBH5 enrichment at two m^6^A peak regions in the *SLC7A5* 3’ UTR of GSCs expressing vector control or RBM12-Flag. n=3.

Data information: Data are shown as mean ± SD. ***P*<0.01, ****P*<0.001, *****P*<0.0001, one-way ANOVA analysis followed by Tukey’s test (B and E), two-way ANOVA analysis followed by Tukey’s test (D), and two-tailed unpaired *t*-test (F).

**Figure S7. ALKBH5 is crucial for GSC proliferation and self-renewal.**

(A) Immunoblot analysis of ALKBH5 expression in five pairs of matched GSCs and NSTCs.

(B) Cell viability assay of GSCs transduced with shNT or shALKBH5. n=4.

(C) EdU incorporation assay of GSCs transduced with shNT or shALKBH5. Scale bar, 60 μm.

(D) Quantification of the fraction of EdU^+^ cells in GSCs expressing shNT or shALKBH5. n=3.

(E) Representative images of tumorspheres derived from GSCs expressing shNT or shALKBH5. Scale bar, 80 μm.

(F and G) Quantification of the diameter (F) and number (G) of tumorspheres derived from GSCs expressing shNT or shALKBH5. n=4.

(H) In vitro limiting dilution assay of the tumorsphere formations of GSCs expressing shNT or shALKBH5.

(I) Immunoblot analysis of pS6, S6, p4E-BP1, 4E-BP1, and ALKBH5 protein levels in GSCs transduced with shNT or shALKBH5.

(J) Immunoblot analysis of SLC7A5, ALKBH5, Myc, and RBM12 protein levels in GSCs transduced with shNT, shRBM12-1, shRBM12-1+Vector, or shRBM12-1+ALKBH5-Myc.

(K) qPCR analysis of *SLC7A5* expression in GSCs transduced with shNT, shRBM12-1, shRBM12-1+Vector, or shRBM12-1+ALKBH5-Myc. n=3.

(L) GSCs expressing shNT, shRBM12-1, shRBM12-1+Vector, or shRBM12-1+ALKBH5-Myc were treated with 9 μg/ml ActD and then harvested at the indicated time points for qPCR analysis. n=3.

Data information: Data are presented as mean ± SD. ***P*<0.01, ****P*<0.001, *****P*<0.0001, two-way ANOVA analysis followed by Tukey’s test (B and L), one-way ANOVA analysis followed by Tukey’s test (D, F, G, and K), and ELDA analysis for differences in stem cell frequencies (H).

**Figure S8. ALKBH5 m^6^A demethylase activity is crucial for *SLC7A5* demethylation and stabilization.**

(A) Immunoblot analysis of SLC7A5 and ALKBH5 expression in GSCs transduced with shNT, shALKBH5-3’ UTR (shALKBH5-3’), shALKBH5-3’+ALKBH5 WT-Myc, or shALKBH5-3’+ALKBH5 H204A-Myc.

(B) qPCR analysis of *SLC7A5* expression in GSCs transduced with shNT, shALKBH5-3’, shALKBH5-3’+ALKBH5 WT-Myc, or shALKBH5-3’+ALKBH5 H204A-Myc. n=3.

(C) GSCs expressing shNT, shALKBH5-3’, shALKBH5-3’+ALKBH5 WT-Myc, or shALKBH5-3’+ALKBH5 H204A-Myc were treated with 9 μg/ml ActD and then harvested at the indicated times for qPCR analysis. n=3.

(D) MeRIP-qPCR analysis of m^6^A enrichment at two m^6^A peak regions in the *SLC7A5* 3’ UTR of GSCs expressing shNT, shALKBH5-3’, shALKBH5-3’+ALKBH5 WT-Myc, or shALKBH5-3’+ALKBH5 H204A-Myc. n=3.

Data information: Data are shown as mean ± SD. ***P*<0.01, ****P*<0.001, *****P*<0.0001, one-way ANOVA analysis followed by Tukey’s test (B and D), and two-way ANOVA analysis followed by Tukey’s test (C). n.s., not significant.

**Figure S9.** **Mutation of residues H34, Q80, or S86 in RBM12** **does not affect RBM12 protein expression, stability or its subcellular localization.**

(A) Immunoblot analysis of Flag expression in GSCs transduced with RBM12 WT-Flag, H34A-Flag, Q80A-Flag, or S86A-Flag.

(B) GSCs expressing RBM12 WT-Flag or H34A-Flag were treated with cycloheximide (CHX, 50 μg/mL) and harvested at the indicated time points for immunoblot analysis.

(C) GSCs expressing RBM12 WT-Flag, Q80A-Flag, or S86A-Flag were treated with CHX (50 μg/mL) and harvested at the indicated time points for immunoblot analysis.

(D) Quantification of Flag levels relative to Tubulin from (B) is shown. n=3

(E) Quantification of Flag levels relative to Tubulin from (C) is shown. n=3

(F) Immunofluorescent staining of Flag (green) in GSCs expressing RBM12 WT-Flag or H34A-Flag. Scale Bar, 30 μm.

(G) Immunofluorescent staining of Flag (green) in GSCs expressing RBM12 WT-Flag, Q80A-Flag, or S86A-Flag. Scale Bar, 30 μm.

Data information: Data are presented as mean ± SD. Two-way ANOVA analysis followed by Sidak's test (D), two-way ANOVA analysis followed by Tukey’s test (E).

**Figure S10. The interaction between RBM12 and ALKBH5 is crucial for *SLC7A5* demethylation and stabilization.**

(A) Immunoblot analysis of SLC7A5, RBM12, and Flag expression in GSCs transduced with shNT, shRBM12-3’ UTR (shRBM12-3’), shRBM12-3’+RBM12 WT-Flag, shRBM12-3’+RBM12 Q80A-Flag, or shRBM12-3’+RBM12 S86A-Flag.

(B) qPCR analysis of *SLC7A5* expression in GSCs transduced with shNT, shRBM12-3’, shRBM12-3’+RBM12 WT-Flag, shRBM12-3’+RBM12 Q80A-Flag, or shRBM12-3’+RBM12 S86A-Flag. n=3.

(C) GSCs expressing shNT, shRBM12-3’, shRBM12-3’+RBM12 WT-Flag, shRBM12-3’+RBM12 Q80A-Flag, or shRBM12-3’+RBM12 S86A-Flag were treated with 9 μg/ml ActD and then harvested at the indicated time points for qPCR analysis. n=3.

(D) RIP-qPCR analysis of ALKBH5 enrichment at two m^6^A peak regions in the *SLC7A5* 3’ UTR of GSCs expressing shNT, shRBM12-3’, shRBM12-3’+RBM12 WT-Flag, or shRBM12-3’+RBM12 H34A-Flag. n=3.

(E) MeRIP-qPCR analysis of m^6^A enrichment at two m^6^A peak regions in the *SLC7A5* 3’ UTR of GSCs expressing shNT, shRBM12-3’, shRBM12-3’+RBM12 WT-Flag, or shRBM12-3’+RBM12 H34A-Flag. n=3.

Data information: Data are shown as mean ± SD. ***P*<0.01, ****P*<0.001, *****P*<0.0001, one-way ANOVA analysis followed by Tukey’s test (B, D, and E), and two-way ANOVA analysis followed by Tukey’s test (C). n.s., not significant.

**Figure S11. OTX1 promotes *RBM12* transcription in GSCs.**

(A) Venn diagram showing the overlap among the following three datasets：the top 20 transcription factors identified via the JASPAR database, genes significantly upregulated in GSCs versus differentiated glioma cells (DGCs) in the GEO dataset GSE247418 (fold change>1.5, *p*<0.05), and genes significantly upregulated in GSCs versus DGCs in the GEO dataset GSE7696 (fold change>1.5, *p*<0.05).

(B) qPCR analysis of *OTX1*, *NFATC2* and *ZBTB12* mRNA expression in GSCs. n=3.

(C) Immunoblot analysis of OTX1 expression in five pairs of matched GSCs and NSTCs.

(D) qPCR analysis of *OTX1* mRNA expression in five pairs of matched GSCs and NSTCs. n=3.

(E) Immunofluorescent staining of OTX1 (green) and SOX2 (red) in human GBM specimens. Scale Bar, 50 μm.

(F) Quantification of the fraction of OTX1^+^ cells in SOX2^+^ cells in three independent human GBMs. n=3.

(G) Immunoblot analysis of RBM12 and OTX1 expression in GSCs transduced with shNT or shOTX1.

(H) qPCR analysis of *RBM12* and *OTX1* mRNA expression in GSCs transduced with shNT or shOTX1. n=3.

(I) Schematic diagram of potential OTX1 binding sites and their mutations in the *RBM12* luciferase reporter.

(J) Reporter gene assays were performed in HEK293T cells transfected with wild-type (WT) *RBM12* promoter reporter (*RBM12-*luc, -2001 to +100), *RBM12*-luc mutant 1 (Site 1 mutation) reporter , *RBM12*-luc mutant 2 (Site 2 mutation) reporter, or *RBM12*-luc mutant 1+2 (Site 1+2 mutations) reporter, in combination with OTX1-Flag or vector control. n=3.

(K) ChlP analysis of OTX1-Flag enrichemnt at two potential OTX1 binding sites of the *RBM12* promoter in GSCs transduced with OTX1-Flag or vector control.

Data information: Data are presented as mean ± SD. ***P*<0.01, ****P*<0.001, *****P*<0.0001, one-way ANOVA analysis followed by Tukey’s test (B and H) and two-tailed unpaired *t*-test (D and J). n.s., not significant.

**Figure S12. JPH203 preferentially targets GSCs and has no effect on body weight in mice with GSC-derived xenografts.**

(A) Dose-response curves for JPH203 in matched GSCs and NSTCs. n=3.

(B) Immunoblot analysis of Cleaved PARP, Cleaved Caspase-7 and RBM12 levels in GSCs treated with the indicated doses of JPH203 or the vehicle control for 48 hours.

(C) Relative body weight of mice bearing GSC (H2S)-derived xenografts treated with JPH203 or the vehicle control at the indicated days. n=9 mice per group.

(D) Relative body weight of mice bearing GSC (T387)-derived xenografts treated with JPH203 or the vehicle control at the indicated days. n=9 mice per group.

Data information: Data are shown as mean ± SD. Two-way ANOVA analysis followed by Sidak's test (C and D).
